# Relationships between housing and management factors and clinical health events in elephants in North American zoos

**DOI:** 10.1371/journal.pone.0217774

**Published:** 2019-06-06

**Authors:** Katie L. Edwards, Michele A. Miller, Kathy Carlstead, Janine L. Brown

**Affiliations:** 1 Center for Species Survival, Smithsonian Conservation Biology Institute, Front Royal, Virginia, United States of America; 2 Department of Science and Technology-National Research Foundation Centre of Excellence for Biomedical TB Research, South African Medical Research Council Centre for Tuberculosis Research, Division of Molecular Biology and Human Genetics, Faculty of Medicine and Health Sciences, Stellenbosch University, Cape Town, South Africa; Centre for Cellular and Molecular Biology, INDIA

## Abstract

Elephants experience a number of health issues that can contribute to their well-being and survival. In managed populations, housing conditions and management practices can influence individual health, so potential risk factors associated with morbidity or mortality should be identified to ensure the best possible standards of care. The goal of this study was to determine if the number of clinical events experienced could be a useful welfare indicator in zoo elephants, and to determine factors associated with key pathologies. We used an epidemiological approach to investigate how intrinsic (species, sex, age) and extrinsic (housing, management) factors were associated with both the total number of clinical events, and each of the four most prevalent pathology types (gastrointestinal issues, skin lesions, lameness, foot lesions), over a 12-month period. The study included 220 (127 African; 93 Asian) elephants housed at 61 facilities across North America. More than 1100 clinical events were identified. Species and sex differences were apparent in the types of pathology encountered, and unsurprisingly, the number of clinical events was positively correlated with age. Factors relating to housing (percent time with indoor/outdoor choice, space experience inside, number of unique environments an elephant was housed in, percent time on soft substrate) and management (enrichment diversity, spread of feeding opportunities) were also related to the number of clinical events. However, relationships were often counter to our initial hypotheses, highlighting caution in assuming cause and effect from correlational analyses such as these. Other welfare indicators such as serum and fecal glucocorticoids and serum prolactin were also associated with health status, being higher or more variable in individuals with a greater number of events. This approach provides insight into housing and management factors related to the health of these species in zoos, and in some cases, may reflect management changes that have already been made to mitigate existing or anticipated health concerns.

## Introduction

In recent years, a scientific approach to studying welfare has led to great strides in improving the care and management of African (*Loxodonta africana*) and Asian (*Elephas maximus*) elephants under human care. In particular, a recent ‘Elephant Welfare Project’ (hereafter EWP) set out to use an epidemiological approach to investigate the factors that impact zoo elephant welfare in North America [1]. That study, conducted by a multi-institutional team of researchers and including 255 elephants at 68 Association of Zoos and Aquariums (AZA) accredited zoos, found that herd social structure, caretaker interactions, and enrichment and feeding diversity correlated with a variety of welfare outcomes [2–11]. In particular, social factors were important for reproductive activity and reducing stereotypic behaviors, training sessions had a positive effect on behavior, and diversity of feeding practices and staff-led exercise decreased the likelihood of an elephant being overweight. Overall, environments that provided diversity and choice were of greater importance to elephant welfare than exhibit size alone, and softer exhibit substrates were beneficial not only for behavioral well-being, but also for foot and joint health.

In addition to the physical [4, 10], behavioral [2, 3, 7], and physiological [5] outcomes measured so far (and reviewed in [12, 13]), health status is another important component of welfare [14], and would benefit from a similar epidemiological approach. While good health does not necessarily equate to preferable welfare, bad health can be an indication of compromised welfare. Suboptimal conditions can increase the risk of injury and/or illness, and prolonged exposure to psychological or physical stressors can result in immunosuppression, decreased wound healing, and increased susceptibility to disease [15].

Elephants experience a range of health issues that contribute to morbidity and mortality. Pathologies such as gastrointestinal issues, foot and joint pathology, skin problems, and dental disorders are relatively common among elephants under human care [16–18], while susceptibility to diseases such as elephant endotheliotropic herpesvirus (EEHV) and tuberculosis (TB) are significant concerns for both *in situ* and *ex situ* populations [19, 20]. Excessive weight and lack of exercise may also contribute to some of these problems [21]. Other conditions, such as foot and joint health [10], injuries [22], and skin lesions [23], could be directly related to extrinsic conditions; and the ability of an individuals’ immune system to combat disease is central to etiopathogenesis. Understanding how different aspects of the captive environment impact health status would be beneficial to overall well-being, and may also aid in our understanding of susceptibility to disease.

Past studies assessing elephant welfare have incorporated health indicators, but have used either discrete assessments of current health status [23, 24], surveys [25], or a review of causes of mortality [21], as opposed to incorporating the total number of active clinical events during a specific study period. The latter approach has, however, been utilized in other species, such as during welfare assessments of laboratory mice [26] and rhesus macaques [27], as well as disease burden in association with stress physiology in captive cheetahs [28], and could be useful in our understanding of how factors impact elephant well-being. The objective of this study was to use the number of clinical events reported for each elephant during the EWP study to assess whether intrinsic (species, sex, age) or extrinsic (environment, management) factors were correlated to this measure of health. This was first conducted considering all clinical events combined, and subsequently focusing on the four most prevalent pathologies. We also set out to assess the relationship between the number of clinical events and other welfare outcome variables from the EWP, such as body condition, cyclicity status, serum cortisol and fecal glucocorticoid metabolite (fGCM) measures, and serum prolactin concentrations. The overall aim was to determine if the number of clinical health events could be a useful indicator of welfare in zoo elephants, and to assess factors associated with key pathologies.

## Methods

### Ethics statement

This research was approved by the management at each participating institution, and where applicable, was reviewed and approved by zoo research committees. In addition, the study protocol was reviewed and approved by the Zoological Society of San Diego Institutional Animal Care and Use Committee (N.I.H. Assurance A3675-01; Protocol 11–203) and the Smithsonian National Zoo (NZP-ACUC #11/10).

### Study population and medical records

This study included elephants housed in Association of Zoos and Aquariums (AZA) accredited zoos during 2012 that were enrolled in the EWP [1]. The species, sex, and age distribution of elephants at the start of the coding period is summarized in Table 1. Twelve consecutive months of medical records were requested for each elephant to coincide with the collection of a variety of independent variables compiled for the EWP. Records were examined and clinical events defined as those that were associated with at least one clinical sign and/or treatment, using a modified coding system from that of Mikota *et al*. [16]. Events were categorized into types and subtypes as defined in Table 2. Preventative treatments, routine vaccinations, and procedures such as ultrasound, artificial insemination, and semen collection were not included. Due to variability between institutions in the reporting within medical records of routine foot care for minor pad/nail defects, foot lesions were only included as events if additional clinical signs were noted alongside a nail crack, or if veterinary treatment was initiated. Similarly, variability between laboratories used for routine serum chemistries and complete blood cell counts (CBCs) meant that abnormal results for these tests were not included as individual events unless associated with at least one clinical sign, or subject to treatment (e.g., anemia). Asymptomatic lab results, such as EEHV viremia or shedding as determined by PCR, reactivity on TB serology testing and fecal parasite burden were included. Chronic conditions were included if they were active (by clinical sign and/or treatment) during the 12-month recording period. Each clinical event was given a score of one; recurrent events were counted individually if they were noted to have resolved and recurred. The duration of medical records reviewed varied slightly between individuals, for example due to births, deaths, and inter-zoo transfers, so duration was also recorded as a variable for use in the analyses, as explained below.

**Table 1:** Species, sex, and age distribution of elephants included in the study (N = 220).

		Age category (years)						
		N	<1	1–5	5–10	10–20	20–40	>40
African	Females	104	2	2	3	2	73	22
	Males	23	2	2	4	3	12	0
Asian	Females	72	2	1	0	5	28	36
	Males	21	1	2	2	3	4	9

**Table 2:** Description of event type and subtype used to categorize clinical data recorded during the 12-month study period in African and Asian elephants in North American zoos.

Event type	Event sub-type	Criteria
Asymptomatic lab result	EEHV	EEHV viremia detected via PCR (either whole blood or trunk wash), but no clinical signs associated with infection
	TB serology	Positive serology test for proteins associated with *Mycobacterium tuberculosis*, but no evidence of *M*. *tuberculosis* on triple trunk-wash culture
	Parasitology	Evidence of parasites (eggs, larvae, bacteria) on fecal examination
	Other	Other lab results that were deemed clinically relevant by the attending veterinarian, but were not associated with clinical signs
Behavioral		Behavioral change noted in the medical record, including anxiety and changes in responsiveness/mental alertness
Death		Death/euthanasia of an elephant
Discharge	Trunk	Discharge from the trunk
	Urogenital	Discharge from the urogenital region
	Unknown source	Discharge from an unknown source or insufficient detail
Ear—irritation/infection		Discharge from or irritation to the ear (excluding the pinnae)
Eaten foreign object		Eaten, or suspected to have eaten, a non-food item, but not associated with gastrointestinal symptoms
Eye	Degenerative	Degenerative changes to the eye
	Injury/irritation	Injury or irritation to the eye
	Unknown cause	Clinical signs associated with the eye, of unknown cause or insufficient detail
Foot lesion		Abscesses, wounds and lesions of the foot, not including nail cracks or pad growth that required no veterinary intervention
Gastrointestinal		Gastrointestinal discomfort, such as colic, bloat and abnormal feces
Idiopathic pain/discomfort		Reported pain or discomfort that could not be otherwise defined
Illness	EEHV	EEHV viremia with additional clinical signs including lethargy, temporal swelling, lameness
	TB	Positive trunk wash culture for *M*. *tuberculosis*, and subsequent treatment for mycobacteriosis
	Other	Signs of illness including lethargy and anorexia, but not otherwise specified
Lameness/stiffness		Lameness, stiffness, decreased range of motion or favoring one or more limbs
Molar disorder		Broken teeth, abnormal tooth growth, swelling or discharge associated with teeth, and any appetite changes considered to be due to dental discomfort
Nutritional/condition		Weight loss, reported loss of condition
Reproductive pathology		Pathological changes to the reproductive tract including cysts and leiomyomas
Respiratory		Abnormal breathing
Skin lesion		Pathological lesions to the skin or oral mucosa, including pustules and abscesses of the skin and hair follicles, pressure sores, dermatitis and hyperkeratosis
Surgery		Surgery and associated wounds
Swelling/mass		Subdermal swelling and masses, not associated with any other clinical sign such as lameness or wounds
Tusk/sulcus injury		Injury to tusk/tush or the sulcus area
Urinary abnormalities		Changes to urination or urine composition
Ventral edema		Accumulation of fluid under the skin, typically on the ventrum or vulva areas
Wound		Injury such as lacerations, abrasions, and fractures, caused by an external factor (object in the environment, conspecific, self-injury)

### Independent variables

Independent variables used for these analyses included a sub-set of the input variables collected during the EWP, and were selected based on hypotheses regarding potential associations with elephant welfare and/or clinical pathologies (Table 3). Full details regarding their collection and calculation are provided in earlier publications [4–6, 8, 11]. In addition, climate zone was assigned to each housing institution based on the National Oceanic and Atmospheric Administration defined nine climatically consistent regions within the contiguous United States [29].

**Table 3:** Description of variables used in the analysis of the number of clinical events recorded during the 12-month study period in African and Asian elephants in North American zoos.

Factor name	Level	Time scale	Description
Identifier	Elephant		A unique identifier for each individual elephant
Zoo	Zoo		A unique identifier for the zoo at which the elephant was housed during the study
Duration	Elephant		Duration of medical record review, in years
Age	Elephant		Age (or estimated age) of the elephant (in years) on the date medical record review started
Sex	Elephant		Male or female
Species	Elephant		African or Asian
Origin	Elephant		Wild born or captive born
Climate zone	Zoo		NOAA US climate zone of the housing facility (Central, East North Central, Northeast, Northwest, South, Southeast, Southwest, West)
Number of transfers^1^	Elephant		The number of facility transfers that an elephant experienced during its lifetime
**Housing variables**^2^			
Herd size	Zoo		Total number of elephants at the housing facility
Environment contact	Elephant	O/D/N	The maximum number of unique environments an elephant was housed in
Animal contact	Elephant	O/D/N	The maximum number of unique elephants that the focal animal was in contact with
Social group contact	Elephant	O/D/N	The maximum number of unique social groups that the focal animal was part of
Space experience:			Average size of the environments (per 1000ft^2^) an elephant spent time in, weighted by the amount of time spent in each environment
Total	Elephant	O/D/N	For all environment types
Indoors	Elephant	O/D/N	For indoor environments only
In/out choice	Elephant	O/D/N	For environments where there is a choice of indoors or outdoors
Outdoors	Elephant	O/D/N	For outdoor environments only
Space experience per elephant	Elephant	O/D/N	Average size of the environments an elephant spends time in, divided by the total number of elephants in the social group using the space at that time, weighted by the amount of time spent in each environment
Relative space experience	Elephant		(Total day space experience—total night space experience)/(total day space experience)
Social experience	Elephant	O/D/N	Average size of the social groups an elephant spent time in, weighted by the amount of time spent in each social group
Relative social experience	Elephant		(Total day social experience—total night social experience)/(total day social experience)
Percent time:			Sum of monthly percent time spent in category, averaged over study period:
Indoors	Elephant	O/D/N	Time spent in indoor environments
Indoor/outdoor choice	Elephant	O/D/N	Time spent in environments with an indoor/outdoor choice
Outdoors	Elephant	O/D/N	Time spent in outdoor environments
Soft substrate	Elephant	O/D/N	Time spent in environment with 100% grass, sand, or rubber substrate
Hard substrate	Elephant	O/D/N	Time spent in environment with 100% concrete or stone aggregate substrate
Time spent with juveniles	Elephant	O/D/N	Time spent in social groups where an elephant 7 years or younger was present
Mixed sex groups	Elephant	O/D/N	Time spent in social groups where both males and females were present
Housed separately	Elephant	O/D/N	Time spent housed alone (social group of 1)
**Management variables**^3^			
Percent time:			Sum of monthly percent time spent in category, averaged over time period:
Managed	Elephant	O/D/N	Percent of time an elephant spent in staff-directed activities, including exercise, husbandry, training time, play, relationship sessions, and demonstrations
Independent	Elephant	O/D/N	Percent of time spent outside of staff-directed activities, including non-managed time on and off exhibit
Enrichment diversity	Zoo		Shannon-Wiener diversity index of the number of enrichment types and frequency with which they were provided
Enrichment program	Zoo		Standardized Factor Score created using a polychoric Principal Components Analysis to examine the frequency of use of the different components of an enrichment program
Feedings	Zoo	O/D/N	Number of feedings during the day, night, or day and night combined
Feeding predictability	Zoo		The predictability of feeding activities
Feed diversity	Zoo		Shannon-Wiener diversity index of the number of feeding types and frequency with which each type was provided
Spread	Zoo		Proportion of all feedings where food was spread throughout the exhibit
Alternative feeding methods	Zoo		Proportion of all feedings where food was presented in a foraging device, hidden, or hung above the exhibit
Exercise per week	Elephant		Number of hours spent exercising each week including walking, stretching, and swimming
Walk per week	Elephant		Number of hours spent walking each week
Exercise diversity	Zoo		Shannon-Wiener diversity index of the number of exercise types and the frequency with which each type was used
**Welfare outcomes**			
BCS^4^	Elephant		Visual assessment of overall body condition, ranging 1–5, with an ideal score being 3
Mean prolactin^5^	Elephant		Serum prolactin concentration, averaged from biweekly serum samples collected across the 12-month study period
Cyclicity status^5^	Elephant		Ovarian cyclicity status based on 12 months of progestogen data
Mean serum cortisol concentration	Elephant		Serum cortisol concentration, averaged from biweekly serum samples collected across the 12-month study period
Serum cortisol SD	Elephant		The standard deviation (SD) calculated from biweekly serum cortisol measurements across the 12-month study period
Serum cortisol CV	Elephant		The coefficient of variation (CV) calculated from biweekly serum cortisol measurements across the 12-month study period
Mean fecal glucocorticoid metabolite concentration	Elephant		Fecal glucocorticoid metabolite concentration, averaged from biweekly serum samples collected across the 12-month study period
Fecal glucocorticoid metabolite concentration SD	Elephant		The standard deviation (SD) calculated from biweekly fecal glucocorticoid metabolite concentration measurements across the 12-month study period
Fecal glucocorticoid metabolite concentration CV	Elephant		The coefficient of variation (CV) calculated from biweekly fecal glucocorticoid metabolite concentration measurements across the 12-month study period

^1^Prado *et al*., 2016

^2^Meehan *et al*., 2016a

^3^Greco *et al*., 2016a

^4^Morfeld *et al*., 2016

^5^Brown *et al*. 2016.

O/D/N: Overall, Day, Night

### Statistical analyses

**Fig 1:**
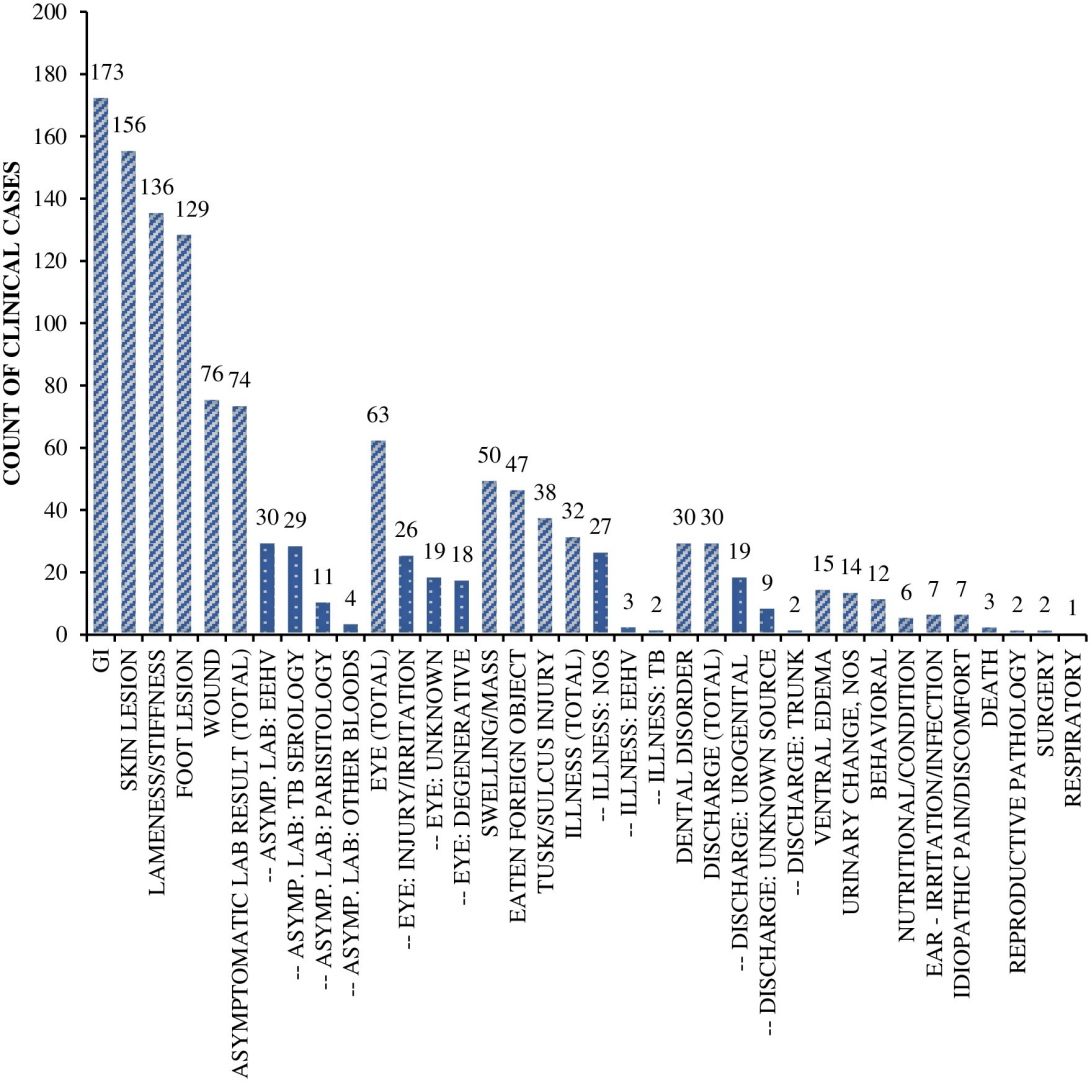
Clinical events by type. Numbers of clinical events (n = 1103) recorded within a 12-month period for 127 African and 93 Asian elephants in North American zoos. Dotted bars represent sub-categories.

Clinical events were categorized by type or sub-type (Fig 1). Data were then analyzed to investigate the relationship between the number of clinical events per individual and EWP independent variables using generalized linear mixed models constructed using the package ‘lmer’ [30] in R [31]. Preliminary univariate analyses were first conducted to assess the relationship between each input variable and the number of clinical events per individual during the study period; any variable that met the criteria of P < 0.15 was taken forward into multivariate analyses. A Poisson distribution with a log-link was used to model the total number of clinical events recorded per individual, with duration of medical records reviewed as an offset variable. Multivariate models incorporated individual and institution as random effects, and age at the start of the coding period as a covariate. For any variables that were determined on different time-scales (i.e. day only [day], night only [night] or over a 24-hr period [overall]), variables for day and night were entered into the same model, but overall variables were not entered along with either day or night. In addition, relationships between the total number of clinical events per individual and each of the nine other welfare outcomes (Table 3) from the EWP were also investigated, with individual and institution as random effects, and duration of medical records reviewed as an offset. These included: BCS [4]; mean prolactin concentration and cyclicity status [5]; and the mean, standard deviation (SD) and coefficient of variation (CV) of both serum cortisol, and fGCM concentration (J. Brown, *unpublished data*).

Models were also created to investigate potential relationships between EWP input variables, or welfare outcomes, with the occurrence of each of the top four most prevalent pathology types. For these subsequent analyses, data were zero-inflated, and so the modelling approach was adjusted. First, a binomial GLMM was used to assess factors associated with the presence or absence of the particular pathology type. Second, a Poisson GLMM with log-link was used as described above to model the number of clinical events, with only individuals that experienced that particular event type included in the model. In both cases, univariate analyses were first conducted based on hypothesized relationships (S1A–S1H Tables), and variables with P < 0.15 used for a multivariate approach.

In all cases, the best fitting model was determined by comparison of the Akaike information criterion (AIC). Collinearity of variables that remained in the final models was also checked using variance inflation factors (VIF), with the criteria that a VIF of 1 indicates no multicollinearity of predictor variables and a VIF of greater than 5 warrants further investigation [32]. VIFs for each variable retained in the final models ranged from 1.00 to 1.32, averaging 1.04, indicating that there was no reason to suspect that results were overly influenced by correlation between the predictor variables in the models. Significance of each variable was assessed using the Wald statistic and chi-squared distribution, with alpha set to 0.05.

## Results

### Clinical events

A total of 1103 clinical events were identified. Out of 220 elephants, only 17 (10 African and 7 Asian) did not exhibit any clinical signs; the remaining 203 elephants experienced between 1 and 22 clinical events during the 12-month study period (median 4 events). These events were categorized into 32 types or sub-types (Table 2; Fig 1), with the most prevalent being gastrointestinal (15.7% of events; 42% of the population), skin lesions (14.1% of events; 37% of the population), lameness and/or stiffness (12.3% of events; 38% of the population), and foot lesions (11.7% of events; 30% of the population).

### Associations between the total number of clinical events and housing and management factors

**Fig 2:**
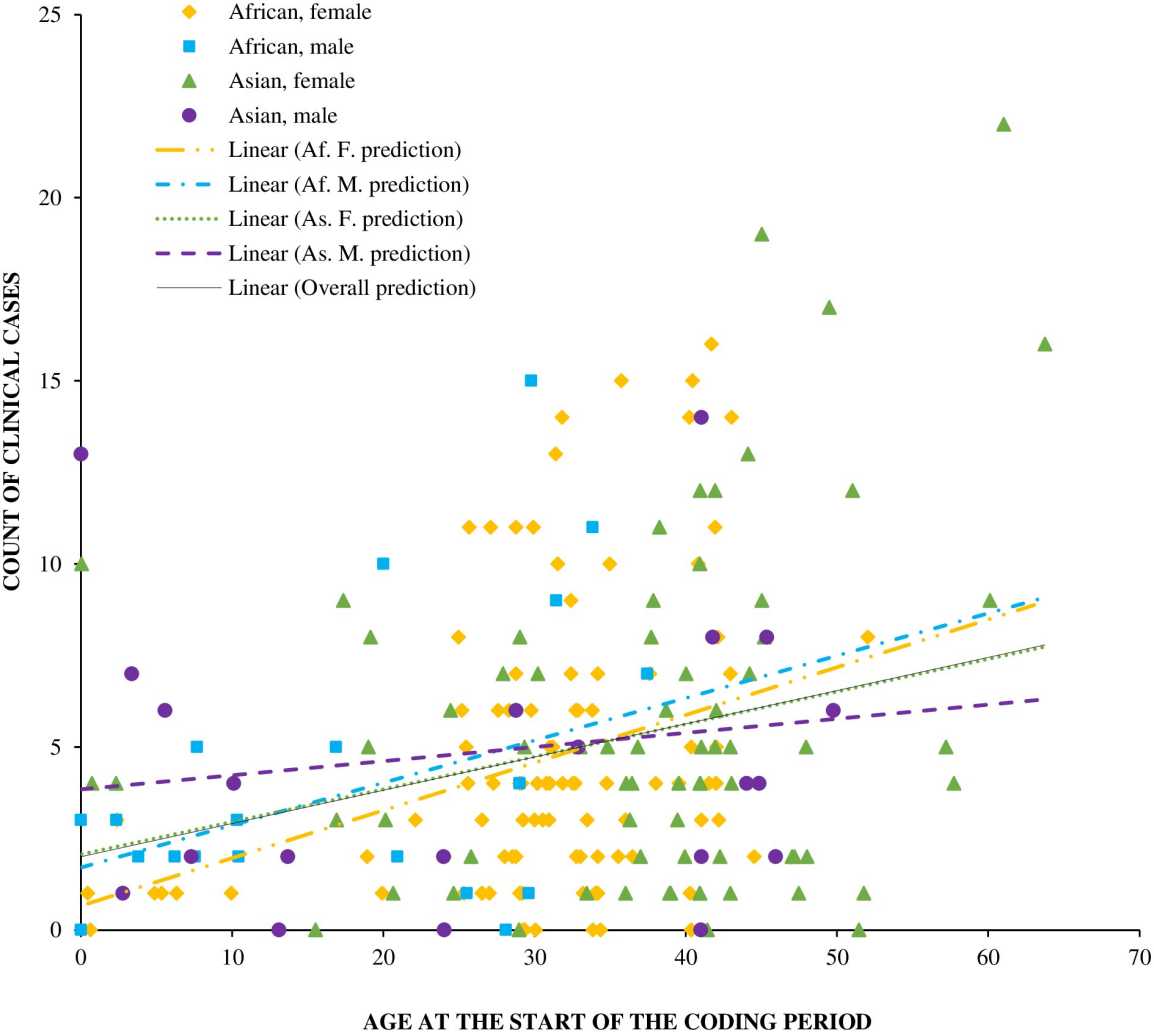
Clinical events by age. The number of clinical events recorded for each individual elephant, plotted according to their age at the start of the recording period. Markers denote individual elephants by species and sex; fit lines represent the GLMM predicted number of clinical events by age according to species, sex and overall.

The results of univariate analyses investigating relationships between housing and management factors and the total number of clinical events per animal are presented in Table 4. These results were used to guide subsequent multivariate analyses (Table 5) and descriptive statistics for variables significant in these models are provided in Table 6. Many of the relationships observed in multivariate models were counter to our original hypotheses, and these are annotated in the respective tables. The best-fit multivariate model revealed that age at the start of the coding period was the strongest predictor of the number of clinical events recorded for an animal (Fig 2), with the number of events increasing by 19% with every additional 10 years of age. This model also included the percent of the daytime that individuals had indoor/outdoor choice, and enrichment diversity; unexpectedly, both being higher in individuals that experienced more clinical events. Based on odds ratios, and keeping all other variables constant, each 10% increase in percent of the daytime spent with indoor/outdoor access choice was associated with 8% greater number of clinical events; an increased enrichment diversity score of 0.1 point was associated with 21% higher rate of clinical events. There was also a tendency for more clinical events in individuals that spent a lower percentage of the night time on hard surfaces, which was opposite to our expectation, and in those that spent a greater proportion of their time housed separately. The number of hours spent walking each week also contributed (positively) to the best-fit model, but did not reach significance.

**Table 4:** Univariate analyses investigating relationships between independent variables and the total number of clinical events per animal in African and Asian elephants in North American zoos. Hypotheses (H): positive (+), negative (-), or neutral (/) relationship of each variable with the number of clinical events per animal as a measure of elephant welfare, and whether observed relationships (P<0.15) were in the predicted direction (Y yes / N no).

Independent variable	Categories	Time scale	H		N	Effect	SE	OR	Wald	df	P
Age			+	Y	212	0.020	0.004	1.021	23.363	1	**0.000**
Sex	Female^†^		/		170						
	Male				42	-0.103	0.137	0.902	0.561	1	0.454
Species	African^†^		/		120						
	Asian				92	0.077	0.157	1.080	0.240	1	0.625
Origin	Wild^†^		/		53						
	Captive			Y^a^	159	-0.423	0.135	0.655	9.813	1	**0.002**
Number of transfers			+		209	0.027	0.028	1.027	0.918	1	0.338
Climate zone	Central^†^		/		45				5.260	7	0.628
	East North Central				2	0.244	0.693	1.277			
	Northeast				19	-0.058	0.306	0.943			
	Northwest				14	0.379	0.350	1.461			
	South				45	0.309	0.232	1.362			
	Southeast				53	0.001	0.251	1.001			
	Southwest				19	0.446	0.313	1.562			
	West				15	0.114	0.332	1.121			
Herd			-		212	-0.032	0.039	0.968	0.677	1	0.411
Environment contact		O	-		212	0.014	0.010	1.014	1.984	1	0.159
		D		N	212	0.020	0.012	1.020	2.678	1	*0*.*102*
		N		N	212	0.024	0.014	1.025	3.151	1	*0*.*076*
Animal contact		O	-		212	-0.025	0.046	0.975	0.301	1	0.583
		D			212	-0.029	0.046	0.971	0.406	1	0.524
		N			212	-0.010	0.052	0.990	0.036	1	0.850
Social group contact		O	/		212	-0.012	0.036	0.988	0.111	1	0.739
		D			212	0.006	0.036	1.006	0.027	1	0.869
		N			212	-0.063	0.064	0.939	0.979	1	0.323
Space experience		O	-		212	-0.002	0.003	0.998	0.425	1	0.515
		D			212	-0.002	0.002	0.998	1.038	1	0.308
		N			212	0.000	0.003	1.000	0.010	1	0.919
Relative space experience			/		212	-0.157	0.161	0.855	0.950	1	0.330
Social experience		O	-		212	-0.088	0.064	0.916	1.880	1	0.170
		D			212	-0.060	0.053	0.941	1.281	1	0.258
		N			212	-0.070	0.064	0.932	1.199	1	0.274
Relative social experience			-		212	-0.119	0.268	0.888	0.196	1	0.658
Percent time indoors		O	+		212	0.000	0.003	1.000	0.001	1	0.974
		D			212	0.003	0.004	1.003	0.652	1	0.419
		N			212	-0.001	0.002	0.999	0.182	1	0.669
Percent time in/out choice		O	-	N	212	0.005	0.003	1.005	2.283	1	*0*.*131*
		D		N	212	0.007	0.004	1.008	4.117	1	**0.042**
		N			212	0.002	0.002	1.002	0.677	1	0.411
Percent time outdoors		O	-		212	-0.004	0.003	0.996	1.984	1	0.159
		D		Y	212	-0.006	0.003	0.994	4.017	1	**0.045**
		N			212	-0.001	0.003	0.999	0.242	1	0.623
Percent time on soft substrate		O	-		212	0.005	0.005	1.005	0.860	1	0.354
		D			212	0.008	0.007	1.008	1.333	1	0.248
		N			212	0.002	0.004	1.002	0.240	1	0.624
Percent time on hard substrate		O	+	N	212	-0.011	0.005	0.990	3.766	1	*0*.*052*
		D			212	-0.015	0.011	0.985	2.049	1	0.152
		N		N	212	-0.008	0.004	0.992	4.901	1	**0.027**
Percent time mixed sex groups		O	-		212	-0.003	0.002	0.997	1.183	1	0.277
		D		Y	212	-0.004	0.002	0.996	3.012	1	*0*.*083*
		N			212	-0.001	0.002	0.999	0.091	1	0.763
Percent time housed separately		O	+	Y	212	0.003	0.002	1.003	2.959	1	*0*.*085*
		D		Y	212	0.003	0.002	1.003	4.513	1	**0.034**
		N			212	0.002	0.001	1.002	1.513	1	0.219
Percent time managed			/		178	0.000	0.004	1.000	0.008	1	0.931
Percent time independent			/		190	-0.002	0.003	0.998	0.483	1	0.487
Enrichment diversity			-	N	199	1.027	0.459	2.792	5.010	1	**0.025**
Enrichment program			-		199	0.005	0.088	1.005	0.004	1	0.952
Feedings		O	-		201	0.017	0.016	1.017	1.155	1	0.283
		D			201	0.028	0.021	1.028	1.803	1	0.179
		N			201	0.006	0.042	1.006	0.021	1	0.884
Feeding predictability			-	N	201	0.234	0.135	1.264	3.013	1	*0*.*083*
Feeding diversity			-	N	201	0.466	0.287	1.593	2.627	1	*0*.*105*
Spread			-	Y	201	-1.151	0.564	0.316	4.175	1	**0.041**
Exercise per week			-		193	0.042	0.044	1.043	0.922	1	0.337
Walk per week			-	N	193	0.094	0.051	1.099	3.369	1	*0*.*066*
Exercise diversity			-		193	0.053	0.145	1.054	0.133	1	0.716

^**†**^Reference category; SE: Standard Error; OR: Odds Ratio; **P < 0.05**; *P > 0*.*15* significance threshold for inclusion in multivariate analysis. O: Overall, D: Day, N: Night.

^a^Wild vs. captive comparison is confounded by age, with captive-origin individuals younger then wild-origin [11].

**Table 5:** Multivariate assessment of the total number of clinical events with housing and management factors by Poisson regression. Hypotheses (H): positive (+) or negative (-) relationship of each variable with the number of clinical events per animal as a measure of elephant welfare, and whether observed relationships were in the predicted direction (Y yes / N no).

Independent variable	H		Effect	SE	OR	Wald	df	P
Intercept			-2.602	1.274	0.074			0.041
Age at the start of the coding period	+	Y	0.019	0.004	1.019	18.431	1	**<0.001**
Percent time with indoor/outdoor choice (day)	-	N	0.008	0.004	1.008	4.675	1	**0.031**
Percent time on hard substrate (night)	+	N	-0.007	0.004	0.993	3.165	1	0.075
Percent time housed separately (day)	+	Y	0.003	0.002	1.003	3.520	1	0.061
Enrichment diversity	-	N	1.131	0.438	3.098	6.669	1	**0.010**
Walk per week	-	N	0.032	0.048	1.032	0.448	1	0.503

SE: Standard Error; OR: Odds Ratio.

**Table 6:** Descriptive statistics (N, mean, standard error of the mean [SEM], minimum and maximum) for significant variables retained in final multivariate models, either for the total number of clinical events per animal (Poisson model), or for the individual number of gastrointestinal, skin lesion, lameness/stiffness and foot lesion event types per animal (binomial and/or Poisson model types).

Independent variable	Model type	Event type	N	Mean	SEM	Min	Max
Age at the start of the coding period*	Poisson	Total number	212	31.35	0.90	0.00	63.76
	Poisson	GI	89	31.35	1.40	0.00	63.76
	Poisson	Skin	77	32.62	1.47	0.72	63.76
	Poisson	Foot	61	34.83	1.27	10.11	61.04
Environment contact (day)	Binomial	GI	212	6.99	0.39	1.00	35.00
Space experience inside per 1000ft (overall)	Poisson	Foot	61	1.46	0.13	0.00	5.00
Percent time with indoor/outdoor choice (day)*	Poisson	Total number	212	9.95	1.29	0.00	89.58
	Binomial	Skin	212	9.95	1.29	0.00	89.58
	Poisson	Skin	77	14.12	2.57	0.00	89.58
Percent time on soft substrate (day)	Poisson	Lameness/stiffness	80	6.76	1.22	0.00	36.87
Enrichment diversity	Poisson	Total number	199	2.86	0.01	2.25	3.27
Spread	Poisson	GI	86	0.24	0.02	0.00	0.71
Body condition score	Binomial	Skin	204	4.05	0.06	1.00	5.00
Mean prolactin concentration*	Poisson	GI	49	18.54	2.67	2.44	105.24
	Poisson	Skin	30	18.50	3.50	4.69	105.24
Serum cortisol concentration: mean (ng/ml)	Poisson	Lameness/stiffness	75	18.79	0.87	5.96	40.02
Serum cortisol concentration: SD*	Poisson	Total number	193	10.08	0.35	0.42	29.81
	Poisson	Lameness/stiffness	74	10.28	0.59	0.42	29.81
Serum cortisol concentration: CV*	Poisson	Total number	193	55.58	1.20	6.84	149.16
	Poisson	GI	80	54.94	2.02	6.84	149.16
Fecal glucocorticoid concentration: mean (ng/g dry feces)	Poisson	Foot	61	103.30	4.17	40.56	205.52
Fecal glucocorticoid concentration: SD	Poisson	Foot	61	34.90	1.99	13.54	85.00

*For variables that remained in multiple models, descriptive statistics are reported for each sample: total number of clinical events and binomial models by subtype utilize the full population; Poisson models by event type utilize only individuals with that event type reported.

### Associations between the total number of clinical events and other welfare outcomes

Models representing univariate analysis of the total number of clinical events per individual with each of the other welfare outcomes from the EWP are presented in Table 7. After adding age at the start of the coding period as a covariate due to the strong relationship with the number of clinical events, only two welfare outcomes were significantly correlated (positive): variability in serum cortisol concentrations, both SD and CV (Table 8). The odds of experiencing a clinical event increased by 24% with a 10 SD increase in serum cortisol, and by 7% for every 10% increase in serum cortisol CV.

**Table 7:** Univariate analyses investigating relationships between welfare outcomes and the total number of clinical events in African and Asian elephants in North American zoos. Hypotheses (H): positive (+), negative (-), or neutral (/) relationship of each variable with the number of clinical events as a measure of elephant welfare, and whether observed relationships (P<0.15) were in the predicted direction (Y yes / N no).

Independent variable	Categories	H		N	Effect	SE	OR	Wald	df	P
Body condition score^1^		+		204	-0.071	0.062		1.279	1	0.258
Mean prolactin concentration^1^		+	Y	92	0.007	0.004	1.008	2.711	1	*0*.*100*
Cyclicity status^2^	Yes^†^			83				3.530	2	0.171
	No	+		60	0.207	0.128	1.244			
	Irregular	+		12	-0.141	0.263	0.878			
Serum cortisol concentration: mean (ng/ml)^3^		+	Y	194	0.015	0.009	1.014	2.737	1	*0*.*098*
Serum cortisol concentration: SD^3^		+	Y	193	0.032	0.012	1.031	7.137	1	**0.008**
Serum cortisol CV^3^		+	Y	193	0.008	0.003	1.008	5.754	1	**0.016**
Fecal glucocorticoid concentration: mean (ng/g dry feces)^3^		+		203	-0.002	0.002	0.999	0.991	1	0.320
Fecal glucocorticoid concentration: SD^3^		+		203	0.001	0.004	1.001	0.025	1	0.874
Fecal glucocorticoid concentration: CV^3^		+	Y	203	0.005	0.001	1.004	18.321	1	**<0.001**

^**†**^Reference category; SE: Standard Error; OR: Odds Ratio; **P < 0.05**; *P > 0*.*15* significance threshold for inclusion in multivariate analysis.

^1^Morfeld *et al*., 2016

^2^Brown *et al*., 2016

^3^Brown, *unpublished*

**Table 8:** Multivariate assessments of the total number of clinical events with the standard deviation^1^ and the coefficient of variation^2^ in serum cortisol by Poisson regression. Hypotheses (H): positive (+) or negative (-) relationship of each variable with the number of clinical events per animal as a measure of elephant welfare, and whether observed relationships were in the predicted direction (Y yes / N no).

Independent variable	H		Effect	SE	OR	Wald	df	P
Intercept^1^			0.226	0.225				0.316
Age at the start of the coding period^1^	+	Y	0.026	0.005	1.026	22.984	1	**<0.001**
Serum cortisol concentration: SD^1^	+	Y	0.024	0.011	1.024	4.649	1	**0.031**
								
Intercept^2^			0.040	0.268				0.883
Age at the start of the coding period^2^	+	Y	0.027	0.005	1.027	24.924	1	**<0.001**
Serum cortisol concentration: CV^2^	+	Y	0.007	0.003	1.007	5.041	1	**0.025**

SE: Standard Error; OR: Odds Ratio.

### Gastrointestinal events

Binomial multivariate regression revealed that Asians were 65% less likely than Africans to experience GI issues (Table 9). In addition, the greater the maximum number of unique environments an elephant was housed in during the day, the more likely it was to experience GI issues; each additional environment was associated with a 7.3% higher odds of experiencing a GI event. As with the total number of clinical events, the number of hours spent walking each week also contributed (positively) to the best-fit model, but did not reach significance. Both of these relationships were counter to our original hypotheses. Including only those elephants that did experience GI events during the study period, a greater number of events was associated with increasing age, and with a reduced proportion of all feedings where food was spread throughout the exhibit (Table 10). There was a 19% increase in the odds of experiencing an additional GI event with each 10 years of age, and the number of GI events experienced decreased by 7.9% with each 0.1 unit increase in spread. Individuals with a greater number of GI events also had higher mean prolactin (OR = 1.011; P = 0.013) and higher variability in serum cortisol (CV; OR = 1.010; P = 0.001). This represents an 11% increase in GI events with every 10 ng/ml in mean prolactin concentration, and a 10% increase in GI events for every 10% increase in serum cortisol CV.

**Table 9:** Multivariate assessment of the occurrence of gastrointestinal events by binomial regression, counting each animal as having experienced (n = 89) or not (n = 123) this type of pathology. Hypotheses (H): positive (+) or negative (-) relationship of each variable with the occurrence of clinical events per animal as a measure of elephant welfare, and whether observed relationships were in the predicted direction (Y yes / N no).

Independent variable	H		Effect	SE	OR	Wald	df	P
Intercept			-0.785	0.397	0.456			0.048
Species (Asian)	/		-1.048	0.374	0.351	7.841	1	**0.005**
Environment contact (day)	-	N	0.070	0.033	1.073	4.669	1	**0.031**
Walk per week	-	N	0.189	0.118	1.208	2.595	1	0.107

SE: Standard Error; OR: Odds Ratio; **P < 0.05**

**Table 10:** Multivariate Poisson regression of factors associated with the number of gastrointestinal events experienced by individual elephants that experienced at least one event (n = 89). Hypotheses (H): positive (+) or negative (-) relationship of each variable with the number of clinical events per animal as a measure of elephant welfare, and whether observed relationships were in the predicted direction (Y yes / N no).

Independent Variable	H		Effect	SE	OR	Wald	df	P
Intercept			0.310	0.281	1.363			0.270
Age at the start of the coding period	+	Y	0.019	0.007	1.019	8.220	1	**0.004**
Spread	-	Y	-1.539	0.649	0.215	5.628	1	**0.018**

SE: Standard Error; OR: Odds Ratio; **P < 0.05**

### Skin lesion events

Older individuals were more likely to experience skin issues, and those with skin issues spent a greater proportion of the daytime with a choice of indoor/outdoor access (Table 11). Each 10% increase in the percent of time provided with a choice of indoor/outdoor access was associated with 24% increase in the odds of an individual experiencing at least one skin lesion event during the study period, which was counter to our hypothesis. In addition, taking into account the effect of age, individuals with skin issues had lower BCS, decreasing the odds of skin events occurring by 28.7% with each unit increase in BCS (Table 12). The total number of skin lesion events experienced was also associated with increasing age, with a 2.6% increase in the number of events with every additional year of age. Each 10% increase in the percentage of the daytime with a choice of indoor/outdoor access was associated with 8% increase in the number of skin lesion events (Table 13). The enrichment program score, which represents the frequency of use of the different components of an enrichment program, also contributed to the final model, but was not a significant factor in predicting the number of skin lesion events experienced during the study period. Finally, individuals with more skin issues had higher mean prolactin, increasing by 15% with every 10 ng/ml increase in mean prolactin concentration (OR = 1.015; P = 0.015).

**Table 11:** Multivariate assessment of the occurrence of skin lesions by binomial regression, counting each animal as having experienced (n = 77) or not (n = 135) this type of pathology. Hypotheses (H): positive (+) or negative (-) relationship of each variable with the occurrence of clinical events per animal as a measure of elephant welfare, and whether observed relationships were in the predicted direction (Y yes / N no).

Independent variable	H		Effect	SE	OR	Wald	df	P
Intercept			-1.844	0.002	0.158			<0.001
Age at the start of the coding period	+	Y	0.025	0.002	1.025	131.160	1	**<0.001**
Percent time with indoor/outdoor choice (day)	-	N	0.023	0.002	1.024	108.200	1	**<0.001**

SE: Standard Error; OR: Odds Ratio; **P < 0.05**

**Table 12:** Multivariate assessment of the occurrence of skin lesions by binomial regression, counting each animal as having experienced (n = 77) or not (n = 135) this type of pathology. Hypotheses (H): positive (+) or negative (-) relationship of each variable with the occurrence of clinical events per animal as a measure of elephant welfare, and whether observed relationships were in the predicted direction (Y yes / N no).

Independent variable	H		Effect	SE	OR	Wald	df	P
Intercept			-0.229	0.003	0.795			<0.001
Age at the start of the coding period	+	Y	0.026	0.002	1.027	122.860	1	**<0.001**
BCS	/		-0.338	0.003	0.713	17618.120	1	**<0.001**

SE: Standard Error; OR: Odds Ratio; **P < 0.05**

**Table 13:** Multivariate Poisson regression of factors associated with the number of skin lesions experienced by individual elephants that experienced at least one event (n = 77). Hypotheses (H): positive (+) or negative (-) relationship of each variable with the number of clinical events per animal as a measure of elephant welfare, and whether observed relationships were in the predicted direction (Y yes / N no).

Independent variable	H		Effect	SE	OR	Wald	df	P
Intercept			-0.408	0.283	0.665			0.149
Age at the start of the coding period	+	Y	0.025	0.007	1.026	11.723	1	**0.001**
Percent time with indoor/outdoor choice (day)	-	N	0.008	0.003	1.008	6.258	1	**0.012**
Enrichment program	-	Y	-0.005	0.093	0.995	0.003	1	0.959

SE: Standard Error; OR: Odds Ratio; **P < 0.05**

### Lameness/stiffness events

Age and sex were the two main factors associated with the occurrence of lameness/stiffness events (Table 14). The odds of experiencing a lameness/stiffness event increased by 6.4% with every year of age; after taking this into account, males had a 270.5% greater odds of experiencing such an event. There was also a tendency for individuals with lameness/stiffness to have a higher score on enrichment diversity. Environment contact, or maximum number of unique environments that an elephant was housed in during a 24-hour period, also contributed (positively) to the best-fit model, but was not significant. Among individuals that experienced events of lameness and/or stiffness, the percent time spent on soft surfaces was a significant factor. Although the mean time spent exclusively on soft substrates during the day was only 6.8% (range 0–36.9%), contrary to our hypothesis, a 10% increase in the percent time on soft substrate was associated with 16% greater number of lameness/stiffness events (Table 15). Finally, individuals with a greater number of lameness/stiffness events had higher mean (OR = 1.033; P = 0.004) and more variable (SD: OR = 1.042; P = 0.011) serum cortisol concentrations. The odds of experiencing an additional lameness/stiffness event increased by 33% with every 10 ng/ml increase in mean cortisol concentration, and by 42% with a 10 SD change in serum cortisol.

**Table 14:** Multivariate assessment of the occurrence of lameness/stiffness by binomial regression, counting each animal as having experienced (n = 80) or not (n = 132) this type of pathology. Hypotheses (H): positive (+) or negative (-) relationship of each variable with the occurrence of clinical events per animal as a measure of elephant welfare, and whether observed relationships were in the predicted direction (Y yes / N no).

Independent variable	H		Effect	SE	OR	Wald	df	P
Intercept			-10.052	3.773	0.000			0.008
Age at the start of the coding period	+	Y	0.062	0.018	1.064	11.750	1	**0.001**
Sex (male)	/		1.310	0.516	3.705	6.442	1	**0.011**
Environment contact (overall)	-	N	0.048	0.031	1.049	2.477	1	0.116
Enrichment diversity	-	N	2.351	1.271	10.494	3.422	1	*0*.*064*

SE: Standard Error; OR: Odds Ratio; **P < 0.05**

**Table 15:** Multivariate Poisson regression of factors associated with the number of lameness/stiffness events experienced by individual elephants that experienced at least one event (n = 80). Hypotheses (H): positive (+) or negative (-) relationship of each variable with the number of clinical events per animal as a measure of elephant welfare, and whether observed relationships were in the predicted direction (Y yes / N no).

Independent variable	H		Effect	SE	OR	Wald	df	P
Intercept			0.264	0.110	1.302			0.017
Percent time on soft substrate (day)	-	N	0.016	0.007	1.016	4.631	1	**0.031**

SE: Standard Error; OR: Odds Ratio; **P < 0.05**

### Foot lesion events

The presence of foot lesions was positively associated with age (Table 16), and for those individuals that experienced foot lesions, the number of pathologies increased by 2.6% for every year of age (Table 17). Counter to our hypothesis, the number of pathologies increased with greater space experience inside across a 24-hour period; every 1000ft increase in space experienced inside was associated with an 18.3% increase in the number of foot lesion events. Taking into account the age effect on foot pathology prevalence, individuals with more foot lesions had higher mean (OR = 1.007; P = 0.015) and variable (SD: OR = 1.011; P = 0.031) fGCMs. The number of foot lesion events increased by 7% with every 10 ng/ml increase in mean fGCMs, and by 11% with a 10 SD increase in in fGCM concentration. Other factors that remained in the best-fit models, but did not reach significance, were: reduced spread of feedings throughout the exhibit, which contributed to presence or absence of foot pathology; and enrichment diversity, which contributed to the number of foot lesion events experienced.

**Table 16:** Multivariate assessment of the occurrence of foot lesions by binomial regression, counting each animal as having experienced (n = 61) or not (n = 151) this type of pathology. Hypotheses (H): positive (+) or negative (-) relationship of each variable with the occurrence of clinical events per animal as a measure of elephant welfare, and whether observed relationships were in the predicted direction (Y yes / N no).

Independent variable	H		Effect	SE	OR	Wald	df	P
Intercept			-1.508	0.619	0.221			0.015
Age at the start of the coding period	+	Y	0.029	0.014	1.029	4.165	1	**0.041**
Spread	-	Y	-1.563	1.243	0.210	1.581	1	0.209

SE: Standard Error; OR: Odds Ratio; **P < 0.05**

**Table 17:** Multivariate Poisson regression of factors associated with the number of foot lesions experienced by individual elephants that experienced at least one event (n = 61). Hypotheses (H): positive (+) or negative (-) relationship of each variable with the number of clinical events per animal as a measure of elephant welfare, and whether observed relationships were in the predicted direction (Y yes / N no).

Independent variable	H		Effect	SE	OR	Wald	df	P
Intercept			-2.730	1.773	0.065			0.124
Age at the start of the coding period	+	Y	0.026	0.010	1.026	7.467	1	**0.006**
Space experience inside per 1000ft (overall)	-	N	0.168	0.084	1.183	3.963	1	**0.047**
Enrichment diversity	-	N	0.737	0.613	2.090	1.445	1	0.229

SE: Standard Error; OR: Odds Ratio; **P < 0.05**

## Discussion

A number of health measures, including foot and joint pathologies [10, 21, 25, 33–35], the presence of wounds and skin lesions [22, 23, 36], and discrete assessments by body region [24], have been used as indicators of elephant well-being under human care, but until now, no studies have assessed the total number of active clinical events during a specific period of time as a potential welfare indicator. Rather than using discrete health assessments [23, 24], surveys sent to veterinarians [25, 37], or a review of causes of morbidity and mortality [21], we created variables based on the total number of clinical events recorded within the 12-month study period of the EWP. Analyses of over 1100 clinical events in 220 elephants revealed that certain pathologies (e.g., gastrointestinal issues, skin lesions, lameness/stiffness, and foot pathologies) varied by species, sex, and age. Furthermore, the number of events experienced by an individual during the study period was also correlated with extrinsic variables relating to housing and management, as well as with other physiological measures of welfare, like serum and fecal glucocorticoids (GC). However, many of the relationships observed during this study, especially those reflecting of housing and management factors, were counter to our initial hypotheses, highlighting that cause and effect cannot be determined from analyses of this kind.

Not surprisingly, when evaluated as the total number of clinical events within the 12-month period, there was a significant positive effect of age. Typically, old age is associated with a decline in health through accumulated somatic damage and immunosuppression [38]. This can result in increased susceptibility to infectious disease [39], decreased rates of wound healing [40] and degenerative conditions such as osteoarthritis [41] and visual impairment [42]. In elephants, age is considered a contributing factor in the pathogenesis of degenerative joint disease [43], foot pathology [44] and reproductive pathologies [45]. Hence, there are specific guidelines for the care of geriatric elephants [46], as pathologies are anticipated to accumulate with increased age.

In this study, after taking age into account, housing and management variables, specifically percent time with indoor/outdoor choice during the daytime, and enrichment diversity, also significantly predicted the total number of clinical events experienced, although not in the predicted direction. Previous results from the EWP highlighted the beneficial effect of choice and outdoor access on behavioral welfare indices. For example, night time stereotypy was lower in individuals that spent more time in environments with both indoor and outdoor access [2], and recumbence was also greater for individuals that had greater outdoor space during the night [7]. Additionally, a case study of three Asian elephants similarly demonstrated a positive impact of providing outdoor access on behavior, with an increase in play behavior and a decrease in swaying [47]. Outdoor access is also important to the welfare of other species. Pigs maintained in outdoor pens had better health indices of welfare compared to those kept indoors [48], and both broiler [49] and laying [50] chickens, and rabbits [51] were more active and expressed more natural behaviors when provided with outdoor pens. Although the positive association between the total number of clinical events and indoor/outdoor access during the day was therefore contrary to our hypothesis, it may be that elephants with a greater number of active pathologies are provided with more choice. This variable reflects management practices of providing choice of indoor/outdoor access to elephants, but does not indicate where elephants were choosing to spend their time. Miller *et al*. [10] also found relationships between foot and joint health and increased indoor/outdoor access that were contrary to their hypothesis, so these findings certainly warrant further investigation at the individual level.

Contrary to our hypothesis, enrichment diversity was also significantly positively associated with the total number of clinical events experienced across the study period. Environmental enrichment has been demonstrated to improve the welfare of a wide range of wildlife species [52], including reducing stereotypies [53–55] and increasing activity levels [56, 57]. These two benefits are of relevance to elephant health, as stereotypies have been proposed as a contributing factor in the development of foot and joint pathologies [35], and increased exercise supports good body condition and GI health [17]. Most of the studies exploring the use of enrichment on elephant behavior have focused on the use of feeding enrichment to increase species-appropriate behaviors, and decrease stereotypies [58–60]. Brown and colleagues [5] found enrichment diversity in the EWP to be positively correlated with reproductive health in African females, both in terms of reduced acyclicity and abnormal secretion of prolactin. However, the relationship between health and enrichment diversity in this study was contrary to our expectation that a greater enrichment diversity score would be correlated with fewer clinical events. The enrichment diversity variable represented the relative frequency and evenness of use of 30 potential enrichment items, ranging from exhibit features such as sand or dirt piles, mud wallows, pools, logs and scratching posts, to the provision of feeding items such as browse and treat boxes/bags, and to additional items or activities used for the purpose of increasing the activity and/or exploration of the elephants’ habitat. However it was beyond the scope of this study to investigate the relative use of each of these individual enrichment types. Our findings may reflect management changes in response to specific clinical events. For example, elephants with dermatitis may be provided with mud wallows, those with hyperkeratosis may be offered items to scratch on, and elephants prone to colic may be given enrichment items to promote exercise. Clinical conditions such as skin lesions can take a relatively long time to resolve, and without more temporal information on when clinical conditions and enrichment occurred, or the types of enrichment used by specific individuals, it is difficult to fully investigate this relationship. Further studies should explore whether certain types of enrichment are more beneficial to physical health, as compared to enrichment primarily intending cognitive stimulation.

The best-fit model explaining the total number of clinical events also included the percent time spent on hard substrate during the night and time housed separately during the day. Although not quite reaching significance, these relationships, negatively and positively associated with the total number of clinical events, respectively, suggest that these two factors may also be important to our overall understanding of factors that impact elephant health and well-being. The negative correlation between time spent on hard substrates and the total number of clinical cases was contrary to our hypothesis, and to previous literature. Hard substrate has been linked to poor welfare in other species, including pigs and cattle [61], greater one-horned rhinoceros [62, 63], and flamingos [64, 65]. Indeed, contrary to evidence that hard surfaces are detrimental to elephant welfare [25, 33, 66], elephants with a greater number of clinical events in our study actually spent less time on hard substrates during the night. One explanation for this unexpected result may be that management changes had already been made to accommodate individuals with these types of clinical conditions. Thus, elephants with multiple health concerns may be provided with softer substrates at night to facilitate sleep, to relieve pre-existing foot or joint conditions, or to ease the development of pressure sores. Elephants with a greater number of active health issues may also be housed separately for periods during the day to facilitate treatments [67]. Factors that contribute to the best-fit model of the total number of clinical events may be useful to increase our understanding on how the captive environment affects overall health and well-being. However, because an epidemiological approach was used, cause and effect relationships still need to be established, and appropriate mitigation strategies for various pathologies should consider elephants individually.

The most common pathology observed in this study was related to GI health, occurring in 42% of the population. This category included diarrhea, constipation, further changes in stool consistency (decreased production, the presence of blood or mucus), bloating, and other signs of abdominal discomfort or colic. Mikota and colleagues [16] also found a high prevalence of GI disorders during a retrospective analysis of health records in North America, totaling 19% of all events compiled, although their categorization of GI pathology also included eating foreign objects and fecal parasitology. For the current study, these events were kept separate if they were asymptomatic; combined they equated to 20.9% of all events, making the total proportion of events similar across the two studies. Suspected causes of GI discomfort included dietary change (especially the source of hay), eating novel food items, ingestion of sand and/or rocks, and decreased exercise. Other potential causes could be related to parasites, salmonellosis, eating foreign objects, and/or toxins [16]. According to Hatt and Clauss [68], the best prophylactic measure for avoiding colic is continuous stimulation of gut peristalsis through feeding high-fiber roughage and minimizing low-fiber foods such as fruits and concentrates. Although the majority of GI events in this study were resolved with or without treatment, since 2001 there have been at least 14 cases of mortality in North America as a result of GI blockage, impaction, damage or twisted gut (M. Miller, *unpublished data*; [69]).

The presence or absence of GI pathology was best explained by a combination of species and the maximum number of unique environments an elephant was housed in during the daytime. African elephants are at a significantly higher risk of experiencing a GI event, which could be due to species-specific differences in the digestive system [70]. Although both species are broadly considered grazers and browsers, digestion coefficients for dry matter, protein, and fiber fractions are higher for Asian compared to African elephants, due to longer digesta retention times [71]. However, zoo diets rarely differentiate forage types fed to the two species [72], and this is certainly an area that warrants further investigation to determine if dietary changes could help reduce GI issues, and indeed whether species differences in susceptibility may be linked to dietary requirements. The fact that elephants with more environment contact (i.e., housed in a greater number of unique environments during the daytime) were also more likely to experience GI issues was contrary to our hypothesis, because a more complex environment is generally thought to elicit more activity and support welfare [73]. Similarly, the number of hours walked per week during staff-led exercise also contributed to the best model of GI occurrence, being higher in individuals with GI pathology, although it did not reach significance. Perhaps this was related to staff making a concerted effort to exercise elephants. The positive correlation between GI events and variables that increase the potential for exercise (environment contact) or direct participation (number of hours spent walking with staff each week) suggest that it may be a common strategy for managing elephants known to be susceptible to GI discomfort. Regular low intensity exercise is generally good for GI mobility in elephants [72] and other species, such as humans and horses [74–76]. Walking had a beneficial effect on body condition in this population [4]; thus, exercise should be employed, not only to mitigate obesity, but perhaps also to promote better digestive health.

We subsequently investigated factors that contributed to a greater number of GI events during the study period, incorporating only those individuals with more than one GI event. Age again was a factor in a greater number of events, as was a reduced proportion of all feedings where food was spread throughout the exhibit. The fact that age was again a contributor to the best model of the number of GI events may be related to decreased walking rates as elephants get older [3], or could be associated with other accumulating health issues such as deteriorating molar condition which could decrease efficiency of food breakdown, leading to GI impaction. Musculoskeletal disorders are also more prevalent in older elephants (this study and [17, 25, 43]), resulting in the administration of NSAIDs that can be prolonged [77]. One of the known side-effects of long-term NSAID use is GI irritation [78–81], and the prophylactic use of GI protectants was common in this study, especially in older elephants. These data suggest that management practices that involve distributing food throughout the elephants’ habitat, as opposed to limiting feedings to a smaller number of areas, may be beneficial to digestive health, again possibly due to increased exercise facilitating good GI mobility.

Skin lesions were also common among this population, with at least one event reported in 37% of elephants, equally distributed between African and Asian, and male and female elephants. Lesions included calluses, dermatitis, hyperkeratosis, folliculitis, ulcers, pustules, skin abscesses, skin irritation, pruritus, and pressure sores. These were distinct from the category ‘wounds’, which were inflicted by conspecifics or by objects in the environment. Although elephant skin is thick, ranging from 1.8 mm on the medial surface of the ear to 3.2 cm on the dorsum [82], it is sensitive, and can take a long time to heal. The finding that a higher BCS is associated with fewer skin lesions may indicate that a certain proportion of these lesions may be caused by pressure between a substrate (like the floor, the wall) and bony structures, with skin being more prone to lesions if this pressure is less absorbed by subcutaneous adipose tissue. Previous reviews have also indicated a high prevalence of skin lesions in captive elephants [16, 17], and consequently skin care is an important aspect of elephant husbandry. In particular, providing bathing, wallowing, and dusting opportunities to promote overall skin health, as well as scratching posts to aid with removal of dead skin build-up are all part of AZA standards of care [46]. The proportion of events attributed to skin issues (14.1% of events) and wounds (6.9% of events) in the current study was lower than that reported by Mikota *et al*. [16], who found nearly a third of all clinical events to be associated with skin wounds and lesions. In the past 20+ years since those data were published, implementation of improved bathing and skin care protocols may have reduced the prevalence of skin lesions in the North American population. Based on our analyses of factors associated with skin pathologies, elephants that experienced lesions were older than those that did not, and spent a greater proportion of the daytime with a choice of indoor/outdoor access. Furthermore, the number of skin events recorded within an individual during the study period also increased with both of these variables. As with several of the other variables discussed, the positive relationship between indoor/outdoor access and skin pathology is contrary to our original hypothesis that choice is beneficial to elephant well-being, and should result in fewer lesions as elephants have more control over their own environment. One alternative, however, is that a choice of indoor/outdoor access is provided in more extreme conditions; i.e., hotter or cooler climates, or at the peak of summer or winter when skin pathology may be more prevalent due to environmental conditions. More detailed investigations might determine when different types of skin pathology are occurring in our captive elephants.

Risk factors associated with musculoskeletal health assessed during a physical examination as part of the EWP, have already been described by Miller *et al*. [10]. They found that only about a quarter of elephants exhibited musculoskeletal pathologies with time on hard substrate and space experienced in indoor/outdoor exhibits contributing to increased risk. Although that study evaluated the same elephants, it was based on a one-time assessment conducted partway through the study. Here, we add to these data by investigating factors associated with the prevalence of lameness, stiffness, or altered range of motion across a 12-month period. Lameness events were reported for elephants of all age-categories, but discriminating between events that were trauma-related versus those of a potentially degenerative nature was beyond the scope of this study due to insufficient detail in medical records. This type of pathology was the third most numerous, representing over 12% of events and occurring in 38% of elephants. The higher rate of events and affected individuals in our study may be due to intermittent or multiple events per individual during the 12-month period, some of which may not have been detected during the single point-in-time examination. When using a multivariate approach to investigate factors associated with the occurrence of lameness, stiffness, or altered range of motion, age and sex were significant predictors of pathology. The significant influence of sex on risk of musculoskeletal pathology is a novel finding compared to Miller *et al*. [10]. In fact, in that dataset, only three out of a possible 36 males that had complete physical exam data were categorized as having musculoskeletal abnormalities, compared to 20 out of 44 with lameness/stiffness in this study. This perhaps indicates that males in this population had intermittent rather than chronic lameness; if so, one-time assessments may not provide a full representation of disease prevalence in that cohort. One possibility for males having more intermittent lameness is that the median age of males in the study was 10 years younger (24 compared to 34 for females). If lameness were more intermittent, and so scored as multiple events, chronic lameness may be essentially underestimated by only receiving a score of 1, even if it were present for the entire 12-month period. When including only those individuals that experienced lameness/stiffness, a greater number of events was associated with spending more time on soft surfaces during the daytime. Although this was also contrary to our hypothesis that softer substrates are beneficial for joint health, it may be another example of where management changes have already been made to accommodate individuals with existing pathologies and prevent disease progression. Previous reports have drawn links between foot and joint pathology and hard surfaces [34]; and indeed, Miller *et al*. [10] found increased risk of musculoskeletal pathology with increasing time on hard surfaces in this same study population. Again, differences in reporting (one-time versus an entire year) may explain why different factors best explained this pathology, but this counter-intuitive result warrants further investigation.

Miller *et al*. [10] also investigated risk factors for individuals experiencing foot lesions, both scored during a one-time physical examination, and those with possible persistent foot pathology—having at least one pathology reported in 2011 and 2012, although not necessarily the same lesion. They found that space experienced during the night, percent time spent on hard substrates and in indoor/outdoor exhibits during the day significantly predicted foot health, along with a tendency for increasing pathology with age. For our study, we analyzed the proportion of all events during the 12-month study period categorized as foot lesions, and found that Asian elephants were at significantly higher risk of experiencing foot lesions than their African counterparts. This corroborates previous analyses [16, 25], where African elephants experienced lower rates of foot pathology than Asians. However, Miller *et al*. [10] found no species difference in the foot score data from their analyses. This discrepancy could be due to differences in categorizing of foot lesions; because different institutions recorded such events differently, we omitted minor foot lesions that did not require veterinary treatment from our investigation, which may have been documented by veterinarians conducting the physical exam. Indeed, only 30% of elephants were considered to have foot lesions according to our criteria, versus 67% during the physical exam [10]. Simple toenail cracks and abnormal wear that only required routine foot care to resolve may therefore have been under-recorded here as compared to Miller *et al*. [10]. If this is the case, perhaps both African and Asian elephants develop foot lesions, but those that require veterinary care are more common among Asians.

To further investigate the factors involved in the occurrence of foot pathology, we first assessed the presence or absence of events. Here, age was the only significant variable, with foot pathologies more likely in older individuals. Because the Asian elephant population is older than the African population [11], this may in part explain the species difference noted in the proportion of events. We subsequently conducted analyses using only those individuals where events were recorded, and found that after taking age into account, the number of foot lesions increased with greater space experienced inside. This variable represents the average size of indoor environments (per 1000ft^2^) an elephant spent time in, weighted by the amount of time spent inside. This variable could therefore reflect that individuals with a greater number of foot lesions spent more of their time inside, and/or those indoor spaces were larger. We have no data on what substrates were present in these spaces specifically, or what individuals were doing while inside, so further investigation into individual-level metrics are necessary to further examine this finding.

Finally, the total number of clinical events, and each of the four most prevalent pathology types, were evaluated in the context of other welfare outcomes used in the EWP. Individuals with a higher total number of clinical events exhibited a higher variability in serum cortisol concentrations, both when assessed as the SD and as the CV. GC measures also were related to several pathology types. The number of lameness/stiffness events were positively associated with mean serum cortisol and SD, and the number of GI events with the CV. Furthermore, fGCM concentrations, both mean and SD, were correlated with the number of foot lesion events. Examples of how GCs may be a useful indicator of health include increased concentrations during sepsis in both humans [83] and horses [84], parvovirus in dogs [85], rheumatoid arthritis [86] and fibromyalgia [87] in humans, and an increased risk of contracting common childhood diseases [88]. Increased variability in GCs has been demonstrated to correlate with abnormal reproductive function, rates of fighting, and institutional mortality rates in rhinoceros [89]. Indeed, GCs have previously been observed to increase during times of illness and injury in elephants (J. Brown, *unpublished data*; [90, 91]), and in other species [92], and these data now suggest that the variability, as well as overall mean concentrations, may be indicators of clinical conditions. When an individual is faced with a potential challenge, including injury or exposure to pathogens, hypothalamic-pituitary-adrenal (HPA) axis activation and secretion of GCs facilitates an immune response [93]. Physiological levels of GCs play an essential role in coping with infection or inflammation [94], but should subsequently be down-regulated to prevent immunosuppressive effects. In individuals with multiple pathologies over time, HPA activation may be more frequent, resulting in greater mean concentrations and variability over time. Alternatively, individuals with underlying disease may in fact be hyper-responsive to additional stressors, resulting in greater increases in circulating GCs, and therefore greater variability [95]. Clinical events associated with lameness/stiffness, and foot lesions, were correlated with both mean concentrations and variability. We were unable to distinguish chronic pathology from acute trauma in this study, so perhaps mean concentrations may be reflective of chronic pathology whereas variability may reflect repeated acute conditions. This subject of individual variation in GC responses to stressors has received more attention in recent years [96], including studies investigating different coping styles and disease susceptibility [97], and examples of infected individuals having higher magnitude responses to subsequent stressors [98]. A better understanding of inter- and intra-individual variation in HPA reactivity, and the implications for health and disease, would be beneficial to our use of GCs as a welfare measure, and incorporating GC analysis as part of routine monitoring could be valuable to detecting sub-clinical pathology before clinical signs are manifest.

Mean prolactin concentrations were also correlated with both the number of GI and skin lesion events. Prolactin is a peptide hormone most commonly known for its role in milk production; however, it is involved in over 300 different processes throughout the body [99]. There is evidence from a variety of species that prolactin influences normal GI function [100], including fluid and electrolyte transport [101, 102], and gut motility [103]. Hyperprolactinemia has also been observed with GI pathology in humans, such as in association with celiac disease activity [104]. The positive relationship between mean prolactin concentration and the number of GI events is an interesting finding that should be explored in more detail. Studies in humans have also revealed a role of prolactin in the pathogenesis of certain skin pathologies [105, 106], with significantly higher serum prolactin in patients with psoriasis, vitiligo and alopecia areata, when compared to controls [107]. The phenomenon of hyperprolactinemia has previously been associated with reproductive dysfunction in African elephants living in North American zoos [108, 109] and these results suggest there may be other impacts on health, which should be explored further.

Although there is much we have learned about elephant biology over the past decades, we still lack a thorough understanding of factors that influence the occurrence of many of the pathologies to which elephants are susceptible. This research highlights the importance of managing elephant health to promote well-being, focusing on common issues that contribute to overall well-being, as well as more widely reported infectious diseases that impact survival. Indeed, the two most prevalent types of clinical events in this study, GI issues and skin lesions, have so far received less attention from a research perspective than foot and musculoskeletal health, which were less prevalent. Species differences in susceptibility to GI issues should be investigated further to determine if dietary needs differ between Asian and African elephants, and if changes could reduce prevalence. Exercise appears to be beneficial, not only to manage body condition [4], but perhaps also to promote better digestive health, and in elephants that are prone to GI issues, increasing the spread of food items around the exhibit could be an effective strategy to reduce the frequency of clinical signs. Skin issues appear to have reduced over the last 20+ years based on comparison of this study to that conducted by Mikota *et al*. [16], but further investigation is needed to understand the apparent positive correlation with choice of indoor/outdoor access.

However, many of our results were not in the hypothesised direction. Of the variables that significantly contributed to one or more best-fit multivariate models, eight factors were significant in the expected direction (age, spread, serum cortisol [mean, SD and CV], fecal GCs [mean and SD], and mean prolactin), while five (percent time with indoor/outdoor choice during the daytime, enrichment diversity, environment contact during the daytime, percent time on soft substrate during the daytime, and space experience inside) were contrary to our expectations. Three others were significant where we had no *a priori* expectation of direction (species, sex and BCS). Of the variables that were in-line with our predictions, the majority were welfare outcomes measured over the same 12-month time period. This may reflect that physiology is a good indicator of current pathology, whereas many of the extrinsic variables measured during the study period may have been changed since the pathologies began to develop, for example in the case of joint pathology that can take decades to develop. One limitation of this type of analysis is that it is correlational in nature, making it important to remember that these findings do not always reflect cause and effect. Rather, they may actually reflect efforts zoos have already taken to mitigate existing or anticipated health concerns. For example, the positive association between time on soft substrates and musculoskeletal health may reflect that changes have already been made to alleviate chronic conditions and reduce further progression. Other factors such as enrichment diversity require further investigation to determine how enrichment items are utilized and what items are most beneficial to physical versus psychological health. Finally, although this population-level approach has highlighted several factors that may impact health and well-being, it is important to monitor the response to management changes at the individual level. By continuing to improve our knowledge of factors that influence elephant health, we can develop mitigation strategies to minimize morbidity and mortality, and ultimately ensure the good welfare of African and Asian elephants in human care.

## Supporting information

S1 Tables Univariate analyses investigating relationships between independent variables and the presence of the four most common pathology types in African and Asian elephants in North American zoos. (DOCX)
