# Supplementary material for: Relationships between housing and management factors and clinical health events in elephants in North American zoos
Source: PLoS One. 2019 Jun 6;14(6):e0217774. doi: 10.1371/journal.pone.0217774 (PMC6553755; doi:10.1371/journal.pone.0217774)
Supplement: S1 Tables — (DOCX) [file pone.0217774.s001.docx]

## **Table A: Univariate binomial analyses investigating relationships between independent variables and the presence of gastrointestinal cases in African and Asian elephants in North American zoos. Hypotheses (H): positive (+) or negative (-) relationship of each variable with the occurrence of clinical events per animal as a measure of elephant welfare, and whether observed relationships were in the predicted direction (Y yes / N no).**

| **Independent Variable** | **Categories** | **Time scale** | **H** | | **N** | **Effect** | **SE** | **OR** | **Wald** | **df** | **P** |
| --- | --- | --- | --- | --- | --- | --- | --- | --- | --- | --- | --- |
| Age |  |  | / |  | 212 | -0.005 | 0.013 | 0.995 | 0.149 | 1 | 0.699 |
| Sex | Female* |  | / |  | 170 |  |  |  |  |  |  |
|  | Male |  |  |  | 42 | -0.207 | 0.394 | 0.813 | 0.275 | 1 | 0.600 |
| Species | African* |  | / |  | 120 |  |  |  |  |  |  |
|  | Asian |  |  |  | 92 | -1.168 | 0.363 | 0.311 | 10.361 | 1 | **0.001** |
| Origin | Wild* |  | / |  | 53 |  |  |  |  |  |  |
|  | Captive |  |  |  | 159 | -0.262 | 0.374 | 0.769 | 0.492 | 1 | 0.483 |
| Climate zone | Central* |  | / |  | 45 |  |  |  | 4.274 | 7 | 0.748 |
|  | East North Central |  |  |  | 2 | 18.172 | 4716.663 | 7.80E+07 |  |  |  |
|  | Northeast |  |  |  | 19 | 0.640 | 0.661 | 1.897 |  |  |  |
|  | Northwest |  |  |  | 14 | 0.129 | 0.764 | 1.138 |  |  |  |
|  | South |  |  |  | 45 | -0.106 | 0.524 | 0.900 |  |  |  |
|  | Southeast |  |  |  | 53 | 0.392 | 0.519 | 1.479 |  |  |  |
|  | Southwest |  |  |  | 19 | 0.693 | 0.683 | 1.999 |  |  |  |
|  | West |  |  |  | 15 | -0.675 | 0.786 | 0.509 |  |  |  |
| Environment contact |  | O | - | N | 212 | 0.056 | 0.027 | 1.058 | 4.145 | 1 | **0.042** |
|  |  | D |  | N | 212 | 0.090 | 0.035 | 1.094 | 6.562 | 1 | **0.010** |
|  |  | N |  | N | 212 | 0.076 | 0.038 | 1.079 | 3.896 | 1 | **0.048** |
| Total space experience |  | O | - | Y | 212 | -0.001 | 0.006 | 0.999 | 0.033 | 1 | 0.855 |
|  |  | D |  | N | 212 | 0.000 | 0.005 | 1.000 | 0.002 | 1 | 0.962 |
|  |  | N |  | N | 212 | 0.000 | 0.007 | 1.000 | 0.000 | 1 | 0.984 |
| Space experience indoors |  | O | + | Y | 212 | 0.127 | 0.127 | 1.135 | 0.993 | 1 | 0.319 |
|  |  | D |  | Y | 212 | 0.103 | 0.116 | 1.108 | 0.793 | 1 | 0.373 |
|  |  | N |  | Y | 212 | 0.159 | 0.126 | 1.172 | 1.579 | 1 | 0.209 |
| Space experience outdoors |  | O | - | N | 212 | 0.000 | 0.005 | 1.000 | 0.008 | 1 | 0.929 |
|  |  | D |  | N | 212 | 0.000 | 0.005 | 1.000 | 0.009 | 1 | 0.924 |
|  |  | N |  | N | 212 | 0.001 | 0.005 | 1.001 | 0.063 | 1 | 0.803 |
| Space experience in/out |  | O | - | Y | 212 | -0.001 | 0.008 | 0.999 | 0.026 | 1 | 0.871 |
|  |  | D |  | N | 212 | 0.001 | 0.008 | 1.001 | 0.021 | 1 | 0.886 |
|  |  | N |  | Y | 212 | -0.001 | 0.007 | 0.999 | 0.016 | 1 | 0.899 |
| Percent time indoors |  | O | + | Y | 212 | 0.005 | 0.008 | 1.005 | 0.368 | 1 | 0.544 |
|  |  | D |  | Y | 212 | 0.001 | 0.010 | 1.001 | 0.016 | 1 | 0.899 |
|  |  | N |  | Y | 212 | 0.003 | 0.006 | 1.003 | 0.325 | 1 | 0.569 |
| Percent time in/out choice |  | O | - | N | 212 | 0.003 | 0.007 | 1.003 | 0.166 | 1 | 0.684 |
|  |  | D |  | N | 212 | 0.013 | 0.009 | 1.013 | 2.107 | 1 | *0.147* |
|  |  | N |  | N | 212 | 0.001 | 0.005 | 1.001 | 0.017 | 1 | 0.897 |
| Percent time outdoors |  | O | - | Y | 212 | -0.005 | 0.007 | 0.995 | 0.599 | 1 | 0.439 |
|  |  | D |  | Y | 212 | -0.007 | 0.007 | 0.993 | 1.248 | 1 | 0.264 |
|  |  | N |  | Y | 212 | -0.004 | 0.006 | 0.996 | 0.502 | 1 | 0.479 |
| Percent time on soft substrate |  | O | - | N | 212 | 0.013 | 0.012 | 1.013 | 1.107 | 1 | 0.293 |
|  |  | D |  | N | 212 | 0.016 | 0.017 | 1.017 | 0.971 | 1 | 0.325 |
|  |  | N |  | N | 212 | 0.013 | 0.010 | 1.013 | 1.635 | 1 | 0.201 |
| Percent time on hard substrate |  | O | + | N | 212 | -0.018 | 0.013 | 0.983 | 1.690 | 1 | 0.194 |
|  |  | D |  | N | 212 | -0.015 | 0.024 | 0.985 | 0.408 | 1 | 0.523 |
|  |  | N |  | N | 212 | -0.008 | 0.009 | 0.992 | 0.702 | 1 | 0.402 |
| Percent time managed |  |  | - | N | 178 | 0.002 | 0.009 | 1.002 | 0.065 | 1 | 0.800 |
| Percent time independent |  |  | / |  | 190 | -0.007 | 0.008 | 0.994 | 0.725 | 1 | 0.395 |
| Enrichment diversity |  |  | - | N | 199 | 0.584 | 1.065 | 1.793 | 0.300 | 1 | 0.584 |
| Enrichment program |  |  | - | N | 199 | 0.178 | 0.212 | 1.194 | 0.699 | 1 | 0.403 |
| Feedings |  | O | - | N | 201 | 0.006 | 0.037 | 1.006 | 0.024 | 1 | 0.876 |
|  |  | D |  | N | 201 | 0.028 | 0.049 | 1.028 | 0.318 | 1 | 0.573 |
|  |  | N |  | Y | 201 | -0.075 | 0.103 | 0.927 | 0.536 | 1 | 0.464 |
| Feeding predictability |  |  | / |  | 201 | 0.547 | 0.334 | 1.727 | 2.685 | 1 | *0.101* |
| Feeding diversity |  |  | - | N | 201 | 0.936 | 0.744 | 2.549 | 1.580 | 1 | 0.209 |
| Spread |  |  | - | N | 201 | 0.132 | 1.331 | 1.141 | 0.010 | 1 | 0.921 |
| Exercise per week |  |  | - | N | 193 | 0.119 | 0.101 | 1.126 | 1.382 | 1 | 0.240 |
| Walk per week |  |  | - | N | 193 | 0.190 | 0.120 | 1.209 | 2.488 | 1 | *0.115* |
| Exercise diversity |  |  | - | N | 193 | 0.081 | 0.330 | 1.085 | 0.061 | 1 | 0.806 |

*Reference category; SE: Standard Error; OR: Odds Ratio. **P < 0.05;** *P > 0.15* significance threshold for inclusion in multivariate analysis.

## **Table B: Univariate Poisson analyses investigating relationships between independent variables and the number of gastrointestinal cases in African and Asian elephants in North American zoos. Hypotheses (H): positive (+) or negative (-) relationship of each variable with the number of clinical events per animal as a measure of elephant welfare, and whether observed relationships were in the predicted direction (Y yes / N no).**

| **Independent variable** | **Categories** | **Time scale** | **H** | | **N** | **Effect** | **SE** | **OR** | **Wald** | **df** | **P** |
| --- | --- | --- | --- | --- | --- | --- | --- | --- | --- | --- | --- |
| Age |  |  | / |  | 89 | 0.019 | 0.002 | 1.019 | 69.485 | 1 | **<0.001** |
| Sex | Female* |  | / |  | 73 |  |  |  |  |  |  |
|  | Male |  |  |  | 16 | -0.246 | 0.247 | 0.782 | 0.990 | 1 | 0.320 |
| Species | African* |  | / |  | 62 |  |  |  |  |  |  |
|  | Asian |  |  |  | 27 | 0.053 | 0.211 | 1.054 | 0.062 | 1 | 0.803 |
| Origin | Wild* |  | / |  | 70 |  |  |  |  |  |  |
|  | Captive |  |  |  | 19 | -0.341 | 0.235 | 0.711 | 2.113 | 1 | *0.146* |
| Climate zone | Central* |  | / |  | 17 |  |  |  | 11.728 | 7 | *0.110* |
|  | East North Central |  |  |  | 2 | -0.214 | 0.798 | 0.808 |  |  |  |
|  | Northeast |  |  |  | 10 | 0.189 | 0.377 | 1.208 |  |  |  |
|  | Northwest |  |  |  | 6 | 0.424 | 0.412 | 1.529 |  |  |  |
|  | South |  |  |  | 16 | 0.632 | 0.298 | 1.881 |  |  |  |
|  | Southeast |  |  |  | 24 | 0.226 | 0.304 | 1.253 |  |  |  |
|  | Southwest |  |  |  | 10 | 0.544 | 0.353 | 1.723 |  |  |  |
|  | West |  |  |  | 4 | 1.150 | 0.414 | 3.159 |  |  |  |
| Environment contact |  | O | - | N | 89 | 0.000 | 0.011 | 1.000 | 0.000 | 1 | 0.993 |
|  |  | D |  | N | 89 | 0.002 | 0.014 | 1.002 | 0.023 | 1 | 0.880 |
|  |  | N |  | N | 89 | 0.004 | 0.016 | 1.004 | 0.060 | 1 | 0.807 |
| Total space experience |  | O | - | Y | 89 | -0.002 | 0.003 | 0.998 | 0.294 | 1 | 0.588 |
|  |  | D |  | Y | 89 | -0.003 | 0.003 | 0.997 | 1.210 | 1 | 0.271 |
|  |  | N |  | N | 89 | 0.000 | 0.003 | 1.000 | 0.009 | 1 | 0.927 |
| Space experience indoors |  | O | + | N | 89 | -0.004 | 0.083 | 0.996 | 0.002 | 1 | 0.961 |
|  |  | D |  | Y | 89 | 0.032 | 0.072 | 1.033 | 0.206 | 1 | 0.650 |
|  |  | N |  | N | 89 | -0.001 | 0.075 | 0.999 | 0.000 | 1 | 0.989 |
| Space experience outdoors |  | O | - | Y | 89 | -0.002 | 0.003 | 0.998 | 0.856 | 1 | 0.355 |
|  |  | D |  | Y | 89 | -0.003 | 0.003 | 0.997 | 1.398 | 1 | 0.237 |
|  |  | N |  | N | 89 | 0.001 | 0.002 | 1.001 | 0.186 | 1 | 0.667 |
| Space experience in/out |  | O | - | N | 89 | 0.000 | 0.004 | 1.000 | 0.004 | 1 | 0.953 |
|  |  | D |  | Y | 89 | -0.003 | 0.005 | 0.997 | 0.406 | 1 | 0.524 |
|  |  | N |  | N | 89 | 0.001 | 0.004 | 1.001 | 0.027 | 1 | 0.870 |
| Percent time indoors |  | O | + | N | 89 | -0.001 | 0.005 | 0.999 | 0.019 | 1 | 0.891 |
|  |  | D |  | Y | 89 | 0.003 | 0.005 | 1.003 | 0.251 | 1 | 0.617 |
|  |  | N |  | N | 89 | -0.002 | 0.003 | 0.998 | 0.231 | 1 | 0.631 |
| Percent time in/out choice |  | O | - | Y | 89 | -0.001 | 0.004 | 0.999 | 0.095 | 1 | 0.758 |
|  |  | D |  | N | 89 | 0.000 | 0.005 | 1.000 | 0.000 | 1 | 0.988 |
|  |  | N |  | Y | 89 | -0.001 | 0.003 | 0.999 | 0.191 | 1 | 0.662 |
| Percent time outdoors |  | O | - | N | 89 | 0.001 | 0.004 | 1.001 | 0.099 | 1 | 0.753 |
|  |  | D |  | Y | 89 | -0.001 | 0.003 | 0.999 | 0.106 | 1 | 0.745 |
|  |  | N |  | N | 89 | 0.003 | 0.003 | 1.003 | 0.832 | 1 | 0.362 |
| Percent time on soft substrate |  | O | - | Y | 89 | -0.001 | 0.006 | 0.999 | 0.034 | 1 | 0.853 |
|  |  | D |  | Y | 89 | -0.003 | 0.009 | 0.997 | 0.090 | 1 | 0.763 |
|  |  | N |  | Y | 89 | -0.002 | 0.005 | 0.998 | 0.132 | 1 | 0.716 |
| Percent time on hard substrate |  | O | + | N | 89 | -0.011 | 0.009 | 0.989 | 1.274 | 1 | 0.259 |
|  |  | D |  | N | 89 | -0.015 | 0.015 | 0.986 | 0.990 | 1 | 0.320 |
|  |  | N |  | N | 89 | -0.006 | 0.005 | 0.994 | 1.292 | 1 | 0.256 |
| Percent time managed |  |  | - | Y | 78 | -0.003 | 0.005 | 0.997 | 0.295 | 1 | 0.587 |
| Percent time independent |  |  | / |  | 83 | 0.001 | 0.004 | 1.001 | 0.055 | 1 | 0.815 |
| Enrichment diversity |  |  | - | Y | 86 | -0.389 | 0.588 | 0.678 | 0.438 | 1 | 0.508 |
| Enrichment program |  |  | - | N | 86 | 0.034 | 0.121 | 1.034 | 0.079 | 1 | 0.779 |
| Feedings |  | O | - | Y | 86 | -0.020 | 0.026 | 0.980 | 0.573 | 1 | 0.449 |
|  |  | D |  | Y | 86 | -0.021 | 0.031 | 0.980 | 0.441 | 1 | 0.507 |
|  |  | N |  | Y | 86 | -0.042 | 0.076 | 0.959 | 0.305 | 1 | 0.581 |
| Feeding predictability |  |  | / |  | 86 | 0.041 | 0.187 | 1.042 | 0.049 | 1 | 0.825 |
| Feeding diversity |  |  | - | Y | 86 | -0.084 | 0.388 | 0.919 | 0.047 | 1 | 0.828 |
| Spread |  |  | - | Y | 86 | -1.581 | 0.704 | 0.206 | 5.044 | 1 | **0.025** |
| Exercise per week |  |  | - | Y | 84 | -0.056 | 0.055 | 0.946 | 1.017 | 1 | 0.313 |
| Walk per week |  |  | - | Y | 84 | -0.049 | 0.062 | 0.953 | 0.612 | 1 | 0.434 |
| Exercise diversity |  |  | - | Y | 84 | -0.145 | 0.186 | 0.865 | 0.607 | 1 | 0.436 |

*Reference category; SE: Standard Error; OR: Odds Ratio. **P < 0.05;** *P > 0.15* significance threshold for inclusion in multivariate analysis.

## **Table C: Univariate binomial analyses investigating relationships between independent variables and the presence of skin lesions in African and Asian elephants in North American zoos. Hypotheses (H): positive (+) or negative (-) relationship of each variable with the occurrence of clinical events per animal as a measure of elephant welfare, and whether observed relationships were in the predicted direction (Y yes / N no).**

| **Independent variable** | **Categories** | **Time scale** | **H** | | **N** | **Effect** | **SE** | **OR** | **Wald** | **df** | **P** |
| --- | --- | --- | --- | --- | --- | --- | --- | --- | --- | --- | --- |
| Age |  |  | / |  | 212 | 0.026 | 0.015 | 1.026 | 3.108 | 1 | *0.078* |
| Sex | Female* |  | / |  | 170 |  |  |  |  |  |  |
|  | Male |  |  |  | 42 | 0.340 | 0.393 | 1.405 | 0.749 | 1 | 0.387 |
| Species | African* |  | / |  | 120 |  |  |  |  |  |  |
|  | Asian |  |  |  | 92 | 0.121 | 0.376 | 1.129 | 0.105 | 1 | 0.747 |
| Origin | Wild* |  | / |  | 53 |  |  |  |  |  |  |
|  | Captive |  |  |  | 159 | -0.733 | 0.428 | 0.481 | 2.936 | 1 | *0.087* |
| Climate zone | Central* |  | / |  | 45 |  |  |  | 4.533 | 7 | 0.717 |
|  | East North Central |  |  |  | 2 | -14.630 | 724.078 | 4.43E-07 |  |  |  |
|  | Northeast |  |  |  | 19 | -1.250 | 0.826 | 0.287 |  |  |  |
|  | Northwest |  |  |  | 14 | 0.006 | 0.815 | 1.006 |  |  |  |
|  | South |  |  |  | 45 | -0.011 | 0.544 | 0.989 |  |  |  |
|  | Southeast |  |  |  | 53 | 0.130 | 0.543 | 1.139 |  |  |  |
|  | Southwest |  |  |  | 19 | 0.690 | 0.707 | 1.994 |  |  |  |
|  | West |  |  |  | 15 | -0.141 | 0.773 | 0.868 |  |  |  |
| Environment contact |  | O | / |  | 212 | 0.023 | 0.025 | 1.023 | 0.850 | 1 | 0.357 |
|  |  | D |  |  | 212 | 0.016 | 0.031 | 1.016 | 0.248 | 1 | 0.619 |
|  |  | N |  |  | 212 | 0.024 | 0.036 | 1.024 | 0.438 | 1 | 0.508 |
| Space experience |  | O | / |  | 212 | -0.005 | 0.007 | 0.995 | 0.475 | 1 | 0.491 |
|  |  | D |  |  | 212 | -0.002 | 0.005 | 0.998 | 0.176 | 1 | 0.675 |
|  |  | N |  |  | 212 | -0.008 | 0.008 | 0.992 | 1.092 | 1 | 0.296 |
| Percent time indoors |  | O | + | N | 212 | -0.003 | 0.008 | 0.997 | 0.135 | 1 | 0.713 |
|  |  | D |  | Y | 212 | 0.005 | 0.010 | 1.005 | 0.279 | 1 | 0.598 |
|  |  | N |  | N | 212 | -0.005 | 0.006 | 0.996 | 0.564 | 1 | 0.453 |
| Percent time in/out choice |  | O | - | N | 212 | 0.018 | 0.008 | 1.019 | 4.977 | 1 | **0.026** |
|  |  | D |  | N | 212 | 0.022 | 0.010 | 1.022 | 5.102 | 1 | **0.024** |
|  |  | N |  | N | 212 | 0.011 | 0.006 | 1.011 | 3.390 | 1 | *0.066* |
| Percent time outdoors |  | O | - | Y | 212 | -0.014 | 0.008 | 0.986 | 2.992 | 1 | *0.084* |
|  |  | D |  | Y | 212 | -0.015 | 0.007 | 0.986 | 4.012 | 1 | **0.045** |
|  |  | N |  | Y | 212 | -0.008 | 0.006 | 0.992 | 1.592 | 1 | 0.207 |
| Percent time managed |  |  | + | N | 178 | -0.005 | 0.009 | 0.995 | 0.311 | 1 | 0.577 |
| Percent time independent |  |  | - | Y | 190 | -0.002 | 0.009 | 0.998 | 0.079 | 1 | 0.779 |
| Enrichment diversity |  |  | - | N | 199 | 0.015 | 1.076 | 1.015 | 0.000 | 1 | 0.989 |
| Enrichment program |  |  | - | Y | 199 | -0.086 | 0.209 | 0.917 | 0.171 | 1 | 0.679 |
| Exercise per week |  |  | - | N | 193 | 0.073 | 0.108 | 1.076 | 0.460 | 1 | 0.498 |
| Walk per week |  |  | / |  | 193 | -0.068 | 0.128 | 0.935 | 0.279 | 1 | 0.597 |
| Exercise diversity |  |  | - | N | 193 | 0.060 | 0.368 | 1.062 | 0.027 | 1 | 0.870 |

*Reference category; SE: Standard Error; OR: Odds Ratio. **P < 0.05;** *P > 0.15* significance threshold for inclusion in multivariate analysis.

## **Table D: Univariate Poisson analyses investigating relationships between independent variables and the number of skin lesions in African and Asian elephants in North American zoos. Hypotheses (H): positive (+) or negative (-) relationship of each variable with the number of clinical events per animal as a measure of elephant welfare, and whether observed relationships were in the predicted direction (Y yes / N no).**

| **Independent variable** | **Categories** | **Time scale** | **H** | | **N** | **Effect** | **SE** | **OR** | **Wald** | **df** | **P** |
| --- | --- | --- | --- | --- | --- | --- | --- | --- | --- | --- | --- |
| Age |  |  | / |  | 77 | 0.024 | 0.007 | 1.025 | 11.303 | 1 | **0.001** |
| Sex | Female* |  | / |  | 59 |  |  |  |  |  |  |
|  | Male |  |  |  | 18 | -0.058 | 0.222 | 0.944 | 0.068 | 1 | 0.795 |
| Species | African* |  | / |  | 42 |  |  |  |  |  |  |
|  | Asian |  |  |  | 35 | -0.046 | 0.199 | 0.955 | 0.052 | 1 | 0.819 |
| Origin | Wild* |  | / |  | 61 |  |  |  |  |  |  |
|  | Captive |  |  |  | 16 | -0.358 | 0.250 | 0.699 | 2.056 | 1 | **0.152** |
| Climate zone | Central* |  | / |  | 16 |  |  |  | 5.278 | 6 | 0.509 |
|  | East North Central |  |  |  | 0 |  |  |  |  |  |  |
|  | Northeast |  |  |  | 3 | 0.283 | 0.463 | 1.327 |  |  |  |
|  | Northwest |  |  |  | 6 | -0.216 | 0.394 | 0.805 |  |  |  |
|  | South |  |  |  | 16 | 0.277 | 0.267 | 1.319 |  |  |  |
|  | Southeast |  |  |  | 21 | -0.110 | 0.271 | 0.896 |  |  |  |
|  | Southwest |  |  |  | 10 | -0.230 | 0.347 | 0.794 |  |  |  |
|  | West |  |  |  | 5 | 0.354 | 0.368 | 1.424 |  |  |  |
| Environment contact |  | O | / |  | 77 | 0.003 | 0.013 | 1.003 | 0.073 | 1 | *0.787* |
|  |  | D |  |  | 77 | 0.015 | 0.015 | 1.015 | 1.020 | 1 | *0.313* |
|  |  | N |  |  | 77 | 0.007 | 0.018 | 1.007 | 0.132 | 1 | *0.716* |
| Space experience |  | O | / |  | 77 | -0.001 | 0.004 | 0.999 | 0.021 | 1 | 0.886 |
|  |  | D |  |  | 77 | -0.002 | 0.003 | 0.998 | 0.413 | 1 | 0.520 |
|  |  | N |  |  | 77 | 0.001 | 0.004 | 1.001 | 0.046 | 1 | 0.830 |
| Percent time indoors |  | O | + | N | 77 | -0.001 | 0.004 | 0.999 | 0.014 | 1 | 0.907 |
|  |  | D |  | Y | 77 | 0.007 | 0.005 | 1.007 | 1.923 | 1 | 0.166 |
|  |  | N |  | N | 77 | -0.003 | 0.003 | 0.997 | 0.686 | 1 | 0.407 |
| Percent time in/out choice |  | O | - | N | 77 | 0.008 | 0.003 | 1.008 | 5.671 | 1 | *0.017* |
|  |  | D |  | N | 77 | 0.009 | 0.003 | 1.009 | 6.847 | 1 | **0.009** |
|  |  | N |  | N | 77 | 0.005 | 0.003 | 1.005 | 3.626 | 1 | 0.057 |
| Percent time outdoors |  | O | - | Y | 77 | -0.008 | 0.003 | 0.992 | 5.097 | 1 | *0.024* |
|  |  | D |  | Y | 77 | -0.009 | 0.003 | 0.991 | 7.595 | 1 | 0.006 |
|  |  | N |  | Y | 77 | -0.004 | 0.003 | 0.996 | 1.513 | 1 | 0.219 |
| Percent time managed |  |  | + | Y | 65 | 0.001 | 0.005 | 1.001 | 0.059 | 1 | 0.809 |
| Percent time independent |  |  | - | Y | 68 | -0.004 | 0.005 | 0.996 | 0.616 | 1 | 0.433 |
| Enrichment diversity |  |  | - | Y | 71 | -0.103 | 0.622 | 0.902 | 0.027 | 1 | **0.869** |
| Enrichment program |  |  | - | Y | 71 | -0.156 | 0.101 | 0.855 | 2.400 | 1 | 0.121 |
| Exercise per week |  |  | - | N | 69 | 0.073 | 0.054 | 1.076 | 1.856 | 1 | 0.173 |
| Walk per week |  |  | / |  | 69 | 0.148 | 0.060 | 1.160 | 6.049 | 1 | *0.014* |
| Exercise diversity |  |  | - | N | 69 | 0.179 | 0.169 | 1.195 | 1.114 | 1 | 0.291 |

*Reference category; SE: Standard Error; OR: Odds Ratio. **P < 0.05;** *P > 0.15* significance threshold for inclusion in multivariate analysis.

## **Table E: Univariate binomial analyses investigating relationships between independent variables and the presence of lameness/stiffness cases in African and Asian elephants in North American zoos. Hypotheses (H): positive (+) or negative (-) relationship of each variable with the occurrence of clinical events per animal as a measure of elephant welfare, and whether observed relationships were in the predicted direction (Y yes / N no).**

| **Independent variable** | **Categories** | **Time scale** | **H** | | **N** | **Effect** | **SE** | **OR** | **Wald** | **df** | **P** |
| --- | --- | --- | --- | --- | --- | --- | --- | --- | --- | --- | --- |
| Age |  |  | + | Y | 212 | 0.036 | 0.014 | 1.037 | 6.537 | 1 | **0.011** |
| Sex | Female* |  | / |  | 170 |  |  |  |  |  |  |
|  | Male |  |  |  | 42 | 0.776 | 0.420 | 2.173 | 3.410 | 1 | *0.065* |
| Species | African* |  | / |  | 120 |  |  |  |  |  |  |
|  | Asian |  |  |  | 92 | -0.048 | 0.381 | 0.953 | 0.016 | 1 | 0.899 |
| Origin | Wild* |  | / |  | 53 |  |  |  |  |  |  |
|  | Captive |  |  |  | 159 | -0.601 | 0.402 | 0.548 | 2.233 | 1 | *0.135* |
| Climate zone | Central* |  | / |  | 45 |  |  |  | 7.739 | 7 | 0.356 |
|  | East North Central |  |  |  | 2 | 22.275 | 512.000 | 4.72E+09 |  |  |  |
|  | Northeast |  |  |  | 19 | -0.575 | 0.739 | 0.563 |  |  |  |
|  | Northwest |  |  |  | 14 | 1.025 | 0.820 | 2.787 |  |  |  |
|  | South |  |  |  | 45 | -0.526 | 0.567 | 0.591 |  |  |  |
|  | Southeast |  |  |  | 53 | 0.156 | 0.563 | 1.169 |  |  |  |
|  | Southwest |  |  |  | 19 | 1.054 | 0.732 | 2.868 |  |  |  |
|  | West |  |  |  | 15 | -0.279 | 0.787 | 0.756 |  |  |  |
| Environment contact |  | O | - | N | 212 | 0.051 | 0.027 | 1.052 | 3.547 | 1 | *0.060* |
|  |  | D |  | N | 212 | 0.059 | 0.032 | 1.061 | 3.304 | 1 | *0.069* |
|  |  | N |  | N | 212 | 0.063 | 0.040 | 1.065 | 2.502 | 1 | *0.114* |
| Space experience |  | O | - | Y | 212 | -0.012 | 0.008 | 0.988 | 2.403 | 1 | *0.121* |
|  |  | D |  | Y | 212 | -0.010 | 0.006 | 0.990 | 2.750 | 1 | *0.097* |
|  |  | N |  | Y | 212 | -0.007 | 0.008 | 0.993 | 0.865 | 1 | 0.352 |
| Percent time indoors |  | O | + | Y | 212 | 0.007 | 0.008 | 1.007 | 0.780 | 1 | 0.377 |
|  |  | D |  | Y | 212 | 0.007 | 0.010 | 1.007 | 0.444 | 1 | 0.505 |
|  |  | N |  | Y | 212 | 0.003 | 0.006 | 1.003 | 0.301 | 1 | 0.583 |
| Percent time in/out choice |  | O | - | N | 212 | 0.000 | 0.008 | 1.000 | 0.001 | 1 | 0.979 |
|  |  | D |  | N | 212 | 0.003 | 0.010 | 1.003 | 0.132 | 1 | 0.717 |
|  |  | N |  | Y | 212 | -0.002 | 0.006 | 0.998 | 0.148 | 1 | 0.701 |
| Percent time outdoors |  | O | - | Y | 212 | -0.006 | 0.007 | 0.994 | 0.671 | 1 | 0.413 |
|  |  | D |  | Y | 212 | -0.005 | 0.007 | 0.995 | 0.573 | 1 | 0.449 |
|  |  | N |  | Y | 212 | -0.001 | 0.006 | 0.999 | 0.021 | 1 | 0.886 |
| Percent time on soft substrate |  | O | - | N | 212 | 0.009 | 0.013 | 1.009 | 0.478 | 1 | 0.490 |
|  |  | D |  | N | 212 | 0.012 | 0.018 | 1.012 | 0.455 | 1 | 0.500 |
|  |  | N |  | N | 212 | 0.004 | 0.011 | 1.004 | 0.151 | 1 | 0.698 |
| Percent time on hard substrate |  | O | + | N | 212 | -0.018 | 0.015 | 0.982 | 1.542 | 1 | 0.214 |
|  |  | D |  | N | 212 | -0.014 | 0.026 | 0.986 | 0.306 | 1 | 0.580 |
|  |  | N |  | N | 212 | -0.008 | 0.010 | 0.992 | 0.757 | 1 | 0.384 |
| Percent time managed |  |  | - | N | 178 | 0.002 | 0.010 | 1.002 | 0.025 | 1 | 0.874 |
| Percent time independent |  |  | / |  | 190 | -0.003 | 0.009 | 0.997 | 0.093 | 1 | 0.760 |
| Enrichment diversity |  |  | - | N | 199 | 2.111 | 1.153 | 8.256 | 3.350 | 1 | *0.067* |
| Enrichment program |  |  | - | N | 199 | 0.090 | 0.220 | 1.095 | 0.169 | 1 | 0.681 |
| Feeding predictability |  |  | - | N | 201 | 0.320 | 0.335 | 1.377 | 0.914 | 1 | 0.339 |
| Spread |  |  | - | Y | 201 | -0.379 | 1.417 | 0.684 | 0.072 | 1 | 0.789 |
| Exercise per week |  |  | - | Y | 193 | -0.081 | 0.119 | 0.923 | 0.462 | 1 | 0.497 |
| Walk per week |  |  | - | Y | 193 | -0.077 | 0.134 | 0.926 | 0.328 | 1 | 0.567 |
| Exercise diversity |  |  | - | Y | 193 | -0.492 | 0.422 | 0.612 | 1.356 | 1 | 0.244 |

*Reference category; SE: Standard Error; OR: Odds Ratio. **P < 0.05;** *P > 0.15* significance threshold for inclusion in multivariate analysis.

## **Table F: Univariate Poisson analyses investigating relationships between independent variables and the number of lameness/stiffness cases in African and Asian elephants in North American zoos. Hypotheses (H): positive (+) or negative (-) relationship of each variable with the number of clinical events per animal as a measure of elephant welfare, and whether observed relationships were in the predicted direction (Y yes / N no).**

| **Independent variable** | **Categories** | **Time scale** | **H** | | **N** | **Effect** | **SE** | **OR** | **Wald** | **df** | **P** |
| --- | --- | --- | --- | --- | --- | --- | --- | --- | --- | --- | --- |
| Age |  |  | + | Y | 80 | 0.002 | 0.007 | 1.002 | 0.097 | 1 | 0.756 |
| Sex | Female* |  | / |  | 60 |  |  |  |  |  |  |
|  | Male |  |  |  | 20 | -0.001 | 0.205 | 0.999 | 0.000 | 1 | 0.995 |
| Species | African* |  | / |  | 44 |  |  |  |  |  |  |
|  | Asian |  |  |  | 36 | -0.064 | 0.180 | 0.938 | 0.125 | 1 | 0.724 |
| Origin | Wild* |  | / |  | 65 |  |  |  |  |  |  |
|  | Captive |  |  |  | 15 | -0.030 | 0.227 | 0.971 | 0.017 | 1 | 0.896 |
| Climate zone | Central* |  | / |  | 17 |  |  |  | 10.448 | 7 | 0.165 |
|  | East North Central |  |  |  | 2 | -0.149 | 0.740 | 0.862 |  |  |  |
|  | Northeast |  |  |  | 5 | 0.807 | 0.353 | 2.241 |  |  |  |
|  | Northwest |  |  |  | 9 | 0.648 | 0.302 | 1.912 |  |  |  |
|  | South |  |  |  | 12 | 0.117 | 0.326 | 1.125 |  |  |  |
|  | Southeast |  |  |  | 19 | 0.100 | 0.296 | 1.105 |  |  |  |
|  | Southwest |  |  |  | 11 | 0.268 | 0.326 | 1.307 |  |  |  |
|  | West |  |  |  | 5 | 0.006 | 0.463 | 1.006 |  |  |  |
| Environment contact |  | O | - | Y | 80 | -0.006 | 0.011 | 0.994 | 0.270 | 1 | 0.603 |
|  |  | D |  | Y | 80 | -0.006 | 0.014 | 0.994 | 0.164 | 1 | 0.685 |
|  |  | N |  | Y | 80 | -0.009 | 0.016 | 0.991 | 0.322 | 1 | 0.571 |
| Space experience |  | O | - | Y | 80 | -0.005 | 0.005 | 0.995 | 1.163 | 1 | 0.281 |
|  |  | D |  | Y | 80 | -0.003 | 0.003 | 0.997 | 0.648 | 1 | 0.421 |
|  |  | N |  | Y | 80 | -0.007 | 0.006 | 0.993 | 1.552 | 1 | 0.213 |
| Percent time indoors |  | O | + | Y | 80 | 0.004 | 0.004 | 1.004 | 0.842 | 1 | 0.359 |
|  |  | D |  | Y | 80 | 0.001 | 0.005 | 1.001 | 0.055 | 1 | 0.815 |
|  |  | N |  | Y | 80 | 0.003 | 0.003 | 1.003 | 1.010 | 1 | 0.315 |
| Percent time in/out choice |  | O | - | Y | 80 | -0.006 | 0.004 | 0.994 | 1.814 | 1 | 0.178 |
|  |  | D |  | Y | 80 | -0.002 | 0.005 | 0.998 | 0.273 | 1 | 0.601 |
|  |  | N |  | Y | 80 | -0.005 | 0.003 | 0.995 | 2.059 | 1 | 0.151 |
| Percent time outdoors |  | O | - | N | 80 | 0.001 | 0.003 | 1.001 | 0.178 | 1 | 0.673 |
|  |  | D |  | N | 80 | 0.001 | 0.004 | 1.001 | 0.081 | 1 | 0.777 |
|  |  | N |  | N | 80 | 0.001 | 0.003 | 1.001 | 0.222 | 1 | 0.638 |
| Percent time on soft substrate |  | O | - | N | 80 | 0.010 | 0.006 | 1.010 | 3.179 | 1 | *0.075* |
|  |  | D |  | N | 80 | 0.016 | 0.007 | 1.016 | 4.631 | 1 | **0.031** |
|  |  | N |  | N | 80 | 0.009 | 0.005 | 1.009 | 3.752 | 1 | *0.053* |
| Percent time on hard substrate |  | O | + | N | 80 | -0.010 | 0.008 | 0.990 | 1.678 | 1 | 0.195 |
|  |  | D |  | N | 80 | -0.016 | 0.013 | 0.984 | 1.555 | 1 | 0.212 |
|  |  | N |  | N | 80 | -0.007 | 0.005 | 0.993 | 2.061 | 1 | 0.151 |
| Percent time managed |  |  | - | N | 65 | 0.003 | 0.005 | 1.003 | 0.517 | 1 | 0.472 |
| Percent time independent |  |  | / |  | 70 | 0.003 | 0.004 | 1.003 | 0.737 | 1 | 0.391 |
| Enrichment diversity |  |  | - | Y | 74 | -0.064 | 0.580 | 0.938 | 0.012 | 1 | 0.912 |
| Enrichment program |  |  | - | N | 74 | 0.094 | 0.096 | 1.099 | 0.968 | 1 | 0.325 |
| Feeding predictability |  |  | - | Y | 75 | -0.001 | 0.151 | 0.999 | 0.000 | 1 | 0.994 |
| Spread |  |  | - | Y | 75 | -0.350 | 0.704 | 0.704 | 0.248 | 1 | 0.619 |
| Exercise per week |  |  | - | N | 71 | 0.042 | 0.059 | 1.042 | 0.492 | 1 | 0.483 |
| Walk per week |  |  | - | N | 71 | 0.073 | 0.061 | 1.075 | 1.405 | 1 | 0.236 |
| Exercise diversity |  |  | - | Y | 71 | -0.010 | 0.170 | 0.990 | 0.003 | 1 | 0.955 |

*Reference category; SE: Standard Error; OR: Odds Ratio. **P < 0.05;** *P > 0.15* significance threshold for inclusion in multivariate analysis.

## **Table G: Univariate binomial analyses investigating relationships between independent variables and the presence of foot lesion cases in African and Asian elephants in North American zoos. Hypotheses (H): positive (+) or negative (-) relationship of each variable with the occurrence of clinical events per animal as a measure of elephant welfare, and whether observed relationships were in the predicted direction (Y yes / N no).**

| **Independent Variable** | **Categories** | **Time Scale** | **H** | | **N** | **Effect** | **SE** | **OR** | **Wald** | **df** | **P** |
| --- | --- | --- | --- | --- | --- | --- | --- | --- | --- | --- | --- |
| Age |  |  | + | Y | 212 | 0.031 | 0.014 | 1.032 | 5.214 | 1 | **0.022** |
| Sex | Female* |  | / |  | 170 |  |  |  |  |  |  |
|  | Male |  |  |  | 42 | -0.740 | 0.455 | 0.477 | 2.645 | 1 | *0.104* |
| Species | African* |  | / |  | 120 |  |  |  |  |  |  |
|  | Asian |  |  |  | 92 | 0.240 | 0.344 | 1.271 | 0.485 | 1 | 0.486 |
| Origin | Wild* |  | / |  | 53 |  |  |  |  |  |  |
|  | Captive |  |  |  | 159 | -0.757 | 0.417 | 0.469 | 3.303 | 1 | *0.069* |
| Climate zone | Central* |  | / |  | 45 |  |  |  | 4.724 | 7 | 0.694 |
|  | East North Central |  |  |  | 2 | 1.052 | 1.537 | 2.864 |  |  |  |
|  | Northeast |  |  |  | 19 | 0.486 | 0.627 | 1.626 |  |  |  |
|  | Northwest |  |  |  | 14 | -0.099 | 0.768 | 0.906 |  |  |  |
|  | South |  |  |  | 45 | 0.189 | 0.502 | 1.208 |  |  |  |
|  | Southeast |  |  |  | 53 | -0.436 | 0.525 | 0.647 |  |  |  |
|  | Southwest |  |  |  | 19 | 0.705 | 0.625 | 2.024 |  |  |  |
|  | West |  |  |  | 15 | 0.270 | 0.696 | 1.310 |  |  |  |
| Environment contact |  | O | - | N | 212 | 0.008 | 0.023 | 1.008 | 0.123 | 1 | 0.726 |
|  |  | D |  | N | 212 | 0.005 | 0.030 | 1.005 | 0.030 | 1 | 0.864 |
|  |  | N |  | N | 212 | 0.030 | 0.034 | 1.030 | 0.800 | 1 | 0.371 |
| Space experience |  | O | - | Y | 212 | -0.005 | 0.007 | 0.995 | 0.574 | 1 | 0.449 |
|  |  | D |  | Y | 212 | -0.005 | 0.005 | 0.995 | 1.053 | 1 | 0.305 |
|  |  | N |  | Y | 212 | -0.002 | 0.007 | 0.998 | 0.075 | 1 | 0.784 |
| Percent time indoors |  | O | + | Y | 212 | 0.004 | 0.008 | 1.004 | 0.291 | 1 | 0.590 |
|  |  | D |  | Y | 212 | 0.009 | 0.009 | 1.009 | 0.856 | 1 | 0.355 |
|  |  | N |  | Y | 212 | 0.002 | 0.006 | 1.002 | 0.111 | 1 | 0.739 |
| Percent time in/out choice |  | O | - | N | 212 | 0.001 | 0.007 | 1.001 | 0.023 | 1 | 0.881 |
|  |  | D |  | N | 212 | 0.008 | 0.008 | 1.009 | 1.008 | 1 | 0.316 |
|  |  | N |  | N | 212 | 0.000 | 0.005 | 1.000 | 0.004 | 1 | 0.950 |
| Percent time outdoors |  | O | - | Y | 212 | -0.005 | 0.006 | 0.995 | 0.557 | 1 | 0.455 |
|  |  | D |  | Y | 212 | -0.008 | 0.006 | 0.992 | 1.775 | 1 | 0.183 |
|  |  | N |  | Y | 212 | -0.002 | 0.005 | 0.998 | 0.160 | 1 | 0.690 |
| Percent time on soft substrate |  | O | - | Y | 212 | -0.001 | 0.012 | 0.999 | 0.012 | 1 | 0.914 |
|  |  | D |  | N | 212 | 0.012 | 0.016 | 1.012 | 0.540 | 1 | 0.463 |
|  |  | N |  | N | 212 | 0.002 | 0.010 | 1.002 | 0.045 | 1 | 0.832 |
| Percent time on hard substrate |  | O | + | Y | 212 | 0.002 | 0.013 | 1.002 | 0.015 | 1 | 0.903 |
|  |  | D |  | N | 212 | -0.013 | 0.023 | 0.987 | 0.301 | 1 | 0.583 |
|  |  | N |  | Y | 212 | 0.002 | 0.009 | 1.002 | 0.039 | 1 | 0.844 |
| Percent time managed |  |  | - | Y | 178 | -0.005 | 0.009 | 0.995 | 0.306 | 1 | 0.581 |
| Percent time independent |  |  | / |  | 190 | 0.003 | 0.008 | 1.003 | 0.103 | 1 | 0.749 |
| Enrichment diversity |  |  | - | N | 199 | 0.686 | 1.006 | 1.986 | 0.465 | 1 | 0.495 |
| Enrichment program |  |  | - | N | 199 | 0.106 | 0.193 | 1.112 | 0.302 | 1 | 0.583 |
| Feeding predictability |  |  | - | N | 201 | 0.152 | 0.291 | 1.164 | 0.272 | 1 | 0.602 |
| Spread |  |  | - | Y | 201 | -1.995 | 1.234 | 0.136 | 2.613 | 1 | *0.106* |
| Exercise per week |  |  | - | N | 193 | 0.140 | 0.102 | 1.151 | 1.886 | 1 | 0.170 |
| Walk per week |  |  | - | N | 193 | 0.057 | 0.119 | 1.059 | 0.232 | 1 | 0.630 |
| Exercise diversity |  |  | - | Y | 193 | -0.052 | 0.364 | 0.949 | 0.020 | 1 | 0.887 |

*Reference category; SE: Standard Error; OR: Odds Ratio. **P < 0.05;** *P > 0.15* significance threshold for inclusion in multivariate analysis.

## **Table H: Univariate Poisson analyses investigating relationships between independent variables and the number of foot lesions in African and Asian elephants in North American zoos. Hypotheses (H): positive (+) or negative (-) relationship of each variable with the number of clinical events per animal as a measure of elephant welfare, and whether observed relationships were in the predicted direction (Y yes / N no).**

| **Independent Variable** | **Categories** | **Time scale** | **H** | | **N** | **Effect** | **SE** | **OR** | **Wald** | **df** | **P** |
| --- | --- | --- | --- | --- | --- | --- | --- | --- | --- | --- | --- |
| Age |  |  | + | Y | 61 | 0.023 | 0.009 | 1.023 | 6.307 | 1 | **0.012** |
| Sex | Female* |  | / |  | 53 |  |  |  |  |  |  |
|  | Male |  |  |  | 8 | 0.238 | 0.262 | 1.268 | 0.823 | 1 | 0.364 |
| Species | African* |  | / |  | 31 |  |  |  |  |  |  |
|  | Asian |  |  |  | 30 | 0.297 | 0.195 | 1.346 | 2.329 | 1 | *0.127* |
| Origin | Wild* |  | / |  | 51 |  |  |  |  |  |  |
|  | Captive |  |  |  | 10 | 0.010 | 0.259 | 1.010 | 0.001 | 1 | 0.970 |
| Climate zone | Central* |  | / |  | 12 |  |  |  | 9.014 | 7 | 0.252 |
|  | East North Central |  |  |  | 1 | 0.552 | 0.753 | 1.737 |  |  |  |
|  | Northeast |  |  |  | 7 | 0.202 | 0.408 | 1.223 |  |  |  |
|  | Northwest |  |  |  | 4 | 0.839 | 0.379 | 2.314 |  |  |  |
|  | South |  |  |  | 14 | 0.675 | 0.311 | 1.965 |  |  |  |
|  | Southeast |  |  |  | 10 | 0.519 | 0.342 | 1.680 |  |  |  |
|  | Southwest |  |  |  | 8 | 0.661 | 0.350 | 1.938 |  |  |  |
|  | West |  |  |  | 5 | 0.020 | 0.483 | 1.020 |  |  |  |
| Environment contact |  | O | - | Y | 61 | -0.002 | 0.014 | 0.998 | 0.026 | 1 | 0.872 |
|  |  | D |  | Y | 61 | -0.004 | 0.019 | 0.996 | 0.050 | 1 | 0.823 |
|  |  | N |  | N | 61 | 0.016 | 0.016 | 1.016 | 0.940 | 1 | 0.332 |
| Space experience |  | O | - | N | 61 | 0.001 | 0.004 | 1.001 | 0.046 | 1 | 0.830 |
|  |  | D |  | N | 61 | 0.000 | 0.003 | 1.000 | 0.012 | 1 | 0.912 |
|  |  | N |  | N | 61 | 0.000 | 0.004 | 1.000 | 0.000 | 1 | 0.987 |
| Percent time indoors |  | O | + | N | 61 | -0.002 | 0.005 | 0.998 | 0.112 | 1 | 0.738 |
|  |  | D |  | Y | 61 | 0.005 | 0.005 | 1.005 | 1.154 | 1 | 0.283 |
|  |  | N |  | N | 61 | -0.003 | 0.003 | 0.997 | 0.986 | 1 | 0.321 |
| Percent time in/out choice |  | O | - | N | 61 | 0.003 | 0.004 | 1.003 | 0.442 | 1 | 0.506 |
|  |  | D |  | Y | 61 | -0.001 | 0.004 | 0.999 | 0.015 | 1 | 0.903 |
|  |  | N |  | N | 61 | 0.002 | 0.003 | 1.002 | 0.438 | 1 | 0.508 |
| Percent time outdoors |  | O | - | Y | 61 | -0.001 | 0.004 | 0.999 | 0.084 | 1 | 0.772 |
|  |  | D |  | Y | 61 | -0.002 | 0.004 | 0.998 | 0.353 | 1 | 0.553 |
|  |  | N |  | N | 61 | 0.001 | 0.003 | 1.001 | 0.081 | 1 | 0.776 |
| Percent time on soft substrate |  | O | - | N | 61 | 0.003 | 0.007 | 1.003 | 0.204 | 1 | 0.652 |
|  |  | D |  | N | 61 | 0.003 | 0.009 | 1.003 | 0.134 | 1 | 0.715 |
|  |  | N |  | N | 61 | 0.001 | 0.006 | 1.001 | 0.023 | 1 | 0.880 |
| Percent time on hard substrate |  | O | + | N | 61 | -0.001 | 0.007 | 0.999 | 0.023 | 1 | 0.880 |
|  |  | D |  | Y | 61 | 0.007 | 0.013 | 1.007 | 0.250 | 1 | 0.617 |
|  |  | N |  | N | 61 | -0.002 | 0.005 | 0.998 | 0.187 | 1 | 0.666 |
| Percent time managed |  |  | - | N | 51 | 0.002 | 0.004 | 1.002 | 0.181 | 1 | 0.670 |
| Percent time independent |  |  | / |  | 55 | -0.002 | 0.004 | 0.998 | 0.227 | 1 | 0.634 |
| Enrichment diversity |  |  | - | N | 58 | 0.984 | 0.625 | 2.674 | 2.476 | 1 | *0.116* |
| Enrichment program |  |  | - | Y | 58 | -0.056 | 0.117 | 0.946 | 0.227 | 1 | 0.634 |
| Feeding predictability |  |  | - | Y | 59 | -0.019 | 0.193 | 0.981 | 0.010 | 1 | 0.922 |
| Spread |  |  | - | Y | 59 | -0.280 | 0.746 | 0.756 | 0.140 | 1 | 0.708 |
| Exercise per week |  |  | - | Y | 56 | -0.087 | 0.068 | 0.917 | 1.655 | 1 | 0.198 |
| Walk per week |  |  | - | N | 56 | 0.021 | 0.074 | 1.021 | 0.083 | 1 | 0.774 |
| Exercise diversity |  |  | - | Y | 56 | -0.065 | 0.195 | 0.937 | 0.112 | 1 | 0.738 |

*Reference category; SE: Standard Error; OR: Odds Ratio. **P < 0.05;** *P > 0.15* significance threshold for inclusion in multivariate analysis.
